# Supplementary material for: Engineering domain-inlaid SaCas9 adenine base editors with reduced RNA off-targets and increased on-target DNA editing
Source: Nat Commun. 2020 Sep 25;11:4871. doi: 10.1038/s41467-020-18715-y (PMC7519688; doi:10.1038/s41467-020-18715-y)

SUPPLEMENTARY INFORMATION

Engineering domain-inlaid SaCas9 adenine base editors with reduced RNA off-targets and increased on-target DNA editing

Minh Thuan Nguyen Tran,[1] Mohd Khairul Nizam Mohd Khalid,[1] Qi Wang,[1] Jacqueline K R Walker,[1] Grace E Lidgerwood,[2,3] Kimberley L Dilworth,[4] Leszek Lisowski,[4,5] Alice Pébay,[2,3] Alex W Hewitt.[1,6]

[1] Menzies Institute for Medical Research, School of Medicine, University of Tasmania, Tasmania, Australia

[2] Department of Surgery, The University of Melbourne, Victoria, Australia

[3] Department of Anatomy and Neuroscience, The University of Melbourne, Victoria, Australia

[4] Translational Vectorology Research Unit, Children's Medical Research Institute, Faculty of Medicine and Health, The University of Sydney, Westmead, Australia

[5] Military Institute of Hygiene and Epidemiology, The Biological Threats Identification and Countermeasure Centre, Puławy, Poland

[6] Centre for Eye Research Australia, The University of Melbourne, Victoria, Australia

Supplementary Table 1
Plasmid description and their corresponding Addgene deposit number for all constructs used in this work.

Plasmid Name	Addgene ID	Description
PX552-CMV-mCherry-U6-SpCas9	87916	sgRNA expressing AAV construct with a mCherry reporter driven by hSyn promoter.
PX552-CMV-mCherry-U6-SaCas9	107053	Backbone vector for cloning in target sgRNA for use with SaCas9
1010-AIDx-CP	135340	Circular permutant of nSpyCas9 at amino acid position 1010 encoding truncated human cytosine deaminase, hAIDx, at the C-terminus.
1029-AIDx-CP	135341	Circular permutant of nSpyCas9 at amino acid position 1029 encoding truncated human cytosine deaminase, hAIDx, at the C-terminus.
1058-AIDx-CP	135342	Circular permutant of nSpyCas9 at amino acid position 1058 encoding truncated human cytosine deaminase, hAIDx, at the C-terminus.
SpyCas9-AIDx	135343	Wildtype nSpyCas9 encoding truncated human cytosine deaminase, hAIDx, at the C-terminus.
AIDx-ID-202	135344	Intradomain insertion of truncated human cytosine deaminase, hAIDx, at position 202 of nSpyCas9
AIDx-ID-208	135345	Intradomain insertion of truncated human cytosine deaminase, hAIDx, at position 208 of nSpyCas9
AIDx-ID-468	135346	Intradomain insertion of truncated human cytosine deaminase, hAIDx, at position 468 of nSpyCas9
AIDx-ID-1058	135347	Intradomain insertion of truncated human cytosine deaminase, hAIDx, at position 1058 of nSpyCas9 with uracil DNA glycosylase inhibitor (UGI) at the C terminus
BE3-NLS-NLS-P2A-BLAST	135348	BE3 (Base editor generation three) with dual C-terminal NLS in a single operon encoding P2A blasticidine resistance
S2 1058 AIDx no UGI	135349	Intradomain insertion of truncated human cytosine deaminase, hAIDx, at position 1058 of nSpyCas9
S2 1058 APOBEC3 no UGI	135350	Intradomain insertion of rat cytosine deaminase, rAPOBEC3, at position 1058 of nSpyCas9
S2 1058 APOBEC3 with UGI	135351	Intradomain insertion of truncated rat cytosine deaminase, rAPOBEC3, at position 1058 of nSpyCas9 with uracil DNA glycosylase inhibitor (UGI) at the C terminus
S1 1029 CP APOBEC3 no UGI	135352	Circular permutant of nSpyCas9 at amino acid position 1029 encoding rat cytosine deaminase, rAPOBEC3 at the N-terminus
S1 1029 CP APOBEC3 with UGI	135353	Circular permutant of nSpyCas9 at amino acid position 1029 encoding rat cytosine deaminase, rAPOBEC3 at the N-terminus with uracil DNA glycosylase inhibitor (UGI) at the C terminus
S1 1029 AIDx with UGI	135354	Circular permutant of nSpyCas9 at amino acid position 1029 encoding truncated human cytosine deaminase, hAIDx, at the C-terminus with uracil DNA glycosylase inhibitor (UGI) at the C terminus
pCMV-ABEMAX-NLS-P2A_BLAST	135355	ABEMax (wildtype Tada monomer coupled with evolved Tada monomer) with dual C-terminal NLS in a single operon encoding P2A blasticidine resistance
S2 1058 ABEMax	135356	Intradomain insertion of ABEMax (wildtype Tada monomer coupled with evolved Tada monomer) at position 1058 of nSpyCas9
S1 1029 CP ABEMax strategy 1	135357	Circular permutant of nSpyCas9 at amino acid position 1029 encoding ABEMax (wildtype Tada monomer coupled with evolved Tada monomer) at the N-terminus
S1 1029 CP ABEMax strategy 2	135358	Circular permutant of nSpyCas9 at amino acid position 1029 encoding de-coupled ABEMax (wildtype Tada monomer at N terminus, evolved Tada monomer at C terminus)
S1 1029 CP ABEMax strategy 3	135359	Circular permutant of nSpyCas9 at amino acid position 1029 encoding ABEMax (wildtype Tada monomer coupled with evolved Tada monomer) at the C-terminus
S1 1029 miniABEMax V82G strategy1	135360	Circular permutant of nSpyCas9 at amino acid position 1029 encoding miniABEMax (evolved Tada monomer with V82G) at the N-terminus
S2 1058 V82G ABEMax	135361	Intradomain insertion of miniABEMax (evolved Tada monomer with V82G) at position 1058 of

		nSpyCas9
miniABEMax V82G wt	135362	nSpyCas9 with N-terminal miniABEMax (V82G)
S1 1029 V82G ABEMax Strategy 3	135363	Circular permutant of nSpyCas9 at amino acid position 1029 encoding miniABEMax (evolved Tada monomer with V82G) at the N-terminus
ABEMax nSaCas9	135364	SaCas9 nickase (D10A) with ABEMax at the N terminus
miniABEMax V82G nSaCas9	135365	SaCas9 nickase (D10A) with single evolved TadA monomer (ABEMax) with a V82G substitution
nSaCas9 D127 miniABEMax V82G	135366	Intradomain insertion of single evolved TadA monomer (V82G) at the specified position (D127)
nSaCas9 D126 miniABEMax V82G	135367	Intradomain insertion of single evolved TadA monomer (V82G) at the specified position (D126)
nSaCas9 E739 miniABEMax V82G	135368	Intradomain insertion of single evolved TadA monomer (V82G) at the specified position (E739)
nSaCas9 E743 miniABEMax V82G	135369	Intradomain insertion of single evolved TadA monomer (V82G) at the specified position (E743)
nSaCas9 E745 miniABEMax V82G	135370	Intradomain insertion of single evolved TadA monomer (V82G) at the specified position (E745)
nSaCas9 F733 miniABEMax V82G	135371	Intradomain insertion of single evolved TadA monomer (V82G) at the specified position (F733)
nSaCas9 N730 miniABEMax V82G	135372	Intradomain insertion of single evolved TadA monomer (V82G) at the specified position (N730)
nSaCas9 E125 miniABEMax V82G	135373	Intradomain insertion of single evolved TadA monomer (V82G) at the specified position (E125)
nSaCas9 E131 miniABEMax V82G	135374	Intradomain insertion of single evolved TadA monomer (V82G) at the specified position (E131)
nSaCas9 L132 miniABEMax V82G	135375	Intradomain insertion of single evolved TadA monomer (V82G) at the specified position (L132)
nSaCas9 N130 miniABEMax V82G	135376	Intradomain insertion of single evolved TadA monomer (V82G) at the specified position (N130)
nSaCas9 E735 miniABEMax V82G	135377	Intradomain insertion of single evolved TadA monomer (V82G) at the specified position (E735)
nSaCas9 K736 miniABEMax V82G	135378	Intradomain insertion of single evolved TadA monomer (V82G) at the specified position (K736)
nSaCas9 I744 miniABEMax V82G	135379	Intradomain insertion of single evolved TadA monomer (V82G) at the specified position (I744)
nSaCas9 G129 miniABEMax V82G	135380	Intradomain insertion of single evolved TadA monomer (V82G) at the specified position (G129)
nSaCas9 H119 miniABEMax V82G	135381	Intradomain insertion of single evolved TadA monomer (V82G) at the specified position (H119)
nSaCas9 M732 miniABEMax V82G	135382	Intradomain insertion of single evolved TadA monomer (V82G) at the specified position (M732)
nSaCas9 M741 miniABEMax V82G	135383	Intradomain insertion of single evolved TadA monomer (V82G) at the specified position (M741)
nSaCas9 N120 miniABEMax V82G	135384	Intradomain insertion of single evolved TadA monomer (V82G) at the specified position (N120)
nSaCas9 P742 miniABEMax V82G	135385	Intradomain insertion of single evolved TadA monomer (V82G) at the specified position (P742)
nSaCas9 Q731 miniABEMax V82G	135386	Intradomain insertion of single evolved TadA monomer (V82G) at the specified position (Q731)
nSaCas9 Q737 miniABEMax V82G	135387	Intradomain insertion of single evolved TadA monomer (V82G) at the specified position (Q737)
nSaCas9 S740 miniABEMax V82G	135388	Intradomain insertion of single evolved TadA monomer (V82G) at the specified position (S740)
nSaCas9 T128 miniABEMax V82G	135389	Intradomain insertion of single evolved TadA monomer (V82G) at the specified position (T128)
nSaCas9 G129 AIDx	140596	Intradomain insertion of hAIDx at the specified position (G129)
nSaCas9 I744 AIDx	140597	Intradomain insertion of hAIDx at the specified position (I744)
nSaCas9 N730 AIDx	140598	Intradomain insertion of hAIDx at the specified position (N730)
Px601 SCP1_nSaCas9_I744_miniABEMax _V82G hU6_sgRNA_site11	140599	AAV construct expressing nSaCas9_I744_miniABEMax_V82G driven by a SCP1 promoter and sgRNA targeting "ABE site 11" driven by a hU6 promoter
Px601 SCP1_nSaCas9_I744_miniABEMax _V82G hU6_sgRNA_LacZ	140600	AAV construct expressing nSaCas9_I744_miniABEMax_V82G driven by a SCP1 promoter and sgRNA targeting LacZ driven by a hU6 promoter

Supplementary Table 2.
Reference sequence of amplicons used in analysis and an overview of oligonucleotide primer sequences for amplicon expansion and cloning methods. sgRNA sequences are also shown with respect to their on-target editing loci.

Amplicon Primer Sequences:

**Description**	**Oligonucleotide Sequence**
CBE-YFP FWD	TCGTCGGCAGCGTCAGATGTGTATAAGAGACAGgcgccaagatccattcgtt
CBE-YFP REV	GTCTCGTGGGCTCGGAGATGTGTATAAGAGACAGcttgccggtggtgcagat
ABE-site16 FWD	TCGTCGGCAGCGTCAGATGTGTATAAGAGACAGttctctatccacctggaatgagttt
ABE-site16 REV	GTCTCGTGGGCTCGGAGATGTGTATAAGAGACAGcagcaatccagcaacacgc
ABE-YFP FWD	TCGTCGGCAGCGTCAGATGTGTATAAGAGACAGcttcttcaagtccgccatgc
ABE-YFP REV	GTCTCGTGGGCTCGGAGATGTGTATAAGAGACAGccttgatgccgttcttctgc
RNCF2 site1 FWD	TCGTCGGCAGCGTCAGATGTGTATAAGAGACAGtgtgcagacaaacggaactcaacc
RNCF2 site1 REV	GTCTCGTGGGCTCGGAGATGTGTATAAGAGACAGccaacatacagaagtcaggaatgcttga
DNAJB1 (RNA site1) FWD	TCGTCGGCAGCGTCAGATGTGTATAAGAGACAGcatggcatgagggtctccatgg
DNAJB1 (RNA site1) REV	GTCTCGTGGGCTCGGAGATGTGTATAAGAGACAGgcgctaccacccggacaag
MTA2 (RNA site2) FWD	TCGTCGGCAGCGTCAGATGTGTATAAGAGACAGgatgtgagggtgcaggtagaggg
MTA2 (RNA site2) REV	GTCTCGTGGGCTCGGAGATGTGTATAAGAGACAGgggattcgttcaagctcacagcc
PTBP2 (RNA site3) FWD	TCGTCGGCAGCGTCAGATGTGTATAAGAGACAGagattttggtaattccccattgcatcg
PTBP2 (RNA site3) REV	GTCTCGTGGGCTCGGAGATGTGTATAAGAGACAGcctgaatagcttcttccactgttgcc
SAP30BP (RNA site4) FWD	TCGTCGGCAGCGTCAGATGTGTATAAGAGACAGcagaacccccctggcagatgttc

SAP30BP (RNA site4) REV	GTCTCGTGGGCTCGGAGATGTGTATAAGAGACAGtcctcagaccagccatgggg
LCMT1 (RNA site5) FWD	TCGTCGGCAGCGTCAGATGTGTATAAGAGACAGattgccaacactcctgatagctgaatg
LCMT1 (RNA site5) REV	GTCTCGTGGGCTCGGAGATGTGTATAAGAGACAGgacttgcaggtctccactcccg
SCAP (RNA site6) FWD	TCGTCGGCAGCGTCAGATGTGTATAAGAGACAGgaagtagacaacacgcagcctcttg
SCAP (RNA site6) REV	GTCTCGTGGGCTCGGAGATGTGTATAAGAGACAGccattgacattcgccggatggag
YFP (CBE-AIDx) FWD	TCGTCGGCAGCGTCAGATGTGTATAAGAGACAGcgcaaatgggcggtaggcgtg
YFP (CBE-AIDx) REV	GTCTCGTGGGCTCGGAGATGTGTATAAGAGACAGgaagtcgtgctgcttcatgtgg
SpCas9 sgRNA YFP ABE top	ACCGgtgaaccgcatcgagctgaa
SpCas9 sgRNA YFP ABE btm	AACttcagctcgatgcggttcacC
SpCas9 ABE16 sgRNA top	ACCGgggaataaatcatagaatcc
SpCas9 ABE16 sgRNA btm	AACggattctatgatttattcccC
SpCas9 RNF2 sgRNA top	ACCGgtcatcttagtcattacctg
SpCas9 RNF2 sgRNA btm	AACcaggtaatgactaagatgacC
SaCas9 YFP sgRNA (SapI) top	ACCGcgcgccaagatccattcgttg
SaCas9 YFP sgRNA (SapI) bottom	AACcaacgaatggatcttggcgcgC
SaCas9 ABE16 sgRNA top (SapI)	ACCGGGACAAGGTTTGAAGGACAGG
SaCas9 ABE ABE 16 sgRNA btm (SapI)	AACCCTGTCCTTCAAACCTTGTCCC
SaCas9 RNCF2 sgRNA top (SapI)	ACCGCATGAAAACTTAAATAGAACA
SaCas9 RNCF2 sgRNA btm (SapI)	AACTGTTCTATTTAAGTTTTCATGC
SaCas9 PCDH15 sgRNA top	ACCGGGTTCaCCTCTCATTCAGATT
SaCas9 PCDH15 sgRNA bottom	AACAATCTGAATGAGAGGtGAACCC
gDNA Off-Target 1 FWD (ABE11)	TCGTCGGCAGCGTCAGATGTGTATAAGAGACAGCAGGCTGCCAACCATACTCA
gDNA Off-Target 1 REV (ABE11)	GTCTCGTGGGCTCGGAGATGTGTATAAGAGACAGgctgattcaagtcaaacc

	acagg
gDNA Off-Target 2 FWD (ABE11)	TCGTCGGCAGCGTCAGATGTGTATAAGAGACAGCCTGCTTACAGGACTTGTGTCCA
gDNA Off-Target 2 REV (ABE11)	GTCTCGTGGGCTCGGAGATGTGTATAAGAGACAGCTGAAAATGGGGCAGTGGAG
gDNA Off-Target 3 FWD (ABE11)	TCGTCGGCAGCGTCAGATGTGTATAAGAGACAGacctctcccactccaggtca
gDNA Off-Target 3 REV (ABE11)	GTCTCGTGGGCTCGGAGATGTGTATAAGAGACAGtgcggatatagcttgccaca
gDNA Off-Target 4 FWD (ABE11)	TCGTCGGCAGCGTCAGATGTGTATAAGAGACAGACCTCTTCGAGTGGGCAGTG
gDNA Off-Target 4 REV (ABE11)	GTCTCGTGGGCTCGGAGATGTGTATAAGAGACAGAAAGACGGTCGAGGTCGTGA
gDNA Off-Target 5 FWD (ABE11)	TCGTCGGCAGCGTCAGATGTGTATAAGAGACAGctgcaagctcaaaaccctga
gDNA Off-Target 5 REV (ABE11)	GTCTCGTGGGCTCGGAGATGTGTATAAGAGACAGGGCATGAGGTGCATTTGGTT
gDNA Off-Target 6 FWD (ABE11)	TCGTCGGCAGCGTCAGATGTGTATAAGAGACAGTCTTGTGCCACTGTGCATGA
gDNA Off-Target 6 REV (ABE11)	GTCTCGTGGGCTCGGAGATGTGTATAAGAGACAGTGCTTCCCCTTCCTGGAATC
gDNA Off-Target 7 FWD (ABE11)	TCGTCGGCAGCGTCAGATGTGTATAAGAGACAGTTGTTCTGCACTTTTTGTTGGAA
gDNA Off-Target 7 REV (ABE11)	GTCTCGTGGGCTCGGAGATGTGTATAAGAGACAGagggagtattgtgcagggcata
gDNA Off-Target 8 FWD (ABE11)	TCGTCGGCAGCGTCAGATGTGTATAAGAGACAGTTCATGAGGAATTTGCAACACAG
gDNA Off-Target 8 REV (ABE11)	GTCTCGTGGGCTCGGAGATGTGTATAAGAGACAGCTCTTGTGGGCAGAATGTCG
gDNA Off-Target 1 FWD (ABE8)	TCGTCGGCAGCGTCAGATGTGTATAAGAGACAGCCTGGGAGTTCCTCCAGACC
gDNA Off-Target 1 REV (ABE8)	GTCTCGTGGGCTCGGAGATGTGTATAAGAGACAGGTGAGCGCTGTGTGTGGGTA
gDNA Off-Target 2 FWD (ABE8)	TCGTCGGCAGCGTCAGATGTGTATAAGAGACAGACTTGGGAAAAGTAAACACTCAAAAA
gDNA Off-Target 2 REV (ABE8)	GTCTCGTGGGCTCGGAGATGTGTATAAGAGACAGTCTTCACCGAACCAGCAGTG
gDNA Off-Target 3 FWD (ABE8)	TCGTCGGCAGCGTCAGATGTGTATAAGAGACAGAAGCTGCGGTGTTGAATGGT
gDNA Off-Target 3 REV (ABE8)	GTCTCGTGGGCTCGGAGATGTGTATAAGAGACAGCACAGCTGCCACTCGCTAAA

gDNA Off-Target 4 FWD (ABE8)	TCGTCGGCAGCGTCAGATGTGTATAAGAGACAGCGGAAATCTTCACCGAACCA
gDNA Off-Target 4 REV (ABE8)	GTCTCGTGGGCTCGGAGATGTGTATAAGAGACAGGGGCTGCTGTCAGGAGAAATC
gDNA Off-Target 5 FWD (ABE8)	TCGTCGGCAGCGTCAGATGTGTATAAGAGACAGTTGGCATTTCCAAATACCCTCTT
gDNA Off-Target 5 REV (ABE8)	GTCTCGTGGGCTCGGAGATGTGTATAAGAGACAGTGAATGCTGGACCAGGAAAG
gDNA Off-Target 6 FWD (ABE8)	TCGTCGGCAGCGTCAGATGTGTATAAGAGACAGcttcctcatgctgcccttct
gDNA Off-Target 6 REV (ABE8)	GTCTCGTGGGCTCGGAGATGTGTATAAGAGACAGtgctcagcagcaatataaacaatga
gDNA Off-Target 7 FWD (ABE8)	TCGTCGGCAGCGTCAGATGTGTATAAGAGACAGtgtccaatgacaggagcaTGA
gDNA Off-Target 7 REV (ABE8)	GTCTCGTGGGCTCGGAGATGTGTATAAGAGACAGGCACAAAAGCCACAAATGGA
gDNA Off-Target 8 FWD (ABE8)	TCGTCGGCAGCGTCAGATGTGTATAAGAGACAGGCCCCTGAGCCATTAAAACC
gDNA Off-Target 8 REV (ABE8)	GTCTCGTGGGCTCGGAGATGTGTATAAGAGACAGAAACAGGTGAGGGCCATGAA
gDNA Off-Target 9 FWD (ABE8)	TCGTCGGCAGCGTCAGATGTGTATAAGAGACAGCAGAGACTCGGGGGACTGAA
gDNA Off-Target 9 REV (ABE8)	GTCTCGTGGGCTCGGAGATGTGTATAAGAGACAGCATGGGTCAAAAGGCAGAGG
gDNA Off-Target 10 FWD (ABE8)	TCGTCGGCAGCGTCAGATGTGTATAAGAGACAGaggcccatatgtgctccaga
gDNA Off-Target 10 REV (ABE8)	GTCTCGTGGGCTCGGAGATGTGTATAAGAGACAGaacagagtttagaaatagccctgcat
gDNA Off-Target 1 FWD (SITE 1)	TCGTCGGCAGCGTCAGATGTGTATAAGAGACAGCCCTGGGCTATTTTTGTCCA
gDNA Off-Target 1 REV (ABE1)	GTCTCGTGGGCTCGGAGATGTGTATAAGAGACAGTGGCCATATTCTCAGCCACA
gDNA Off-Target 2 FWD (ABE1)	TCGTCGGCAGCGTCAGATGTGTATAAGAGACAGAGTCCCGACAGGCTTTCCAT
gDNA Off-Target 2 REV (ABE1)	GTCTCGTGGGCTCGGAGATGTGTATAAGAGACAGAGATGGGCTTGAGCGATTCA
gDNA Off-Target 3 FWD (ABE1)	TCGTCGGCAGCGTCAGATGTGTATAAGAGACAGTGCCCTTATGAAGAGAAGGAGGA
gDNA Off-Target 3 REV (ABE1)	GTCTCGTGGGCTCGGAGATGTGTATAAGAGACAGTTGGGTCCCTAAGCCAATCC

gDNA Off-Target 4 FWD (ABE1)	TCGTCGGCAGCGTCAGATGTGTATAAGAGACAGTGAAAGCTAGGCATGGGTGAA
gDNA Off-Target 4 REV (ABE1)	GTCTCGTGGGCTCGGAGATGTGTATAAGAGACAGcattcccaaggtcccagaaa
gDNA Off-Target 5 FWD (ABE1)	TCGTCGGCAGCGTCAGATGTGTATAAGAGACAGTGAGCATTTCTCTCAGGTGTCC
gDNA Off-Target 5 REV (ABE1)	GTCTCGTGGGCTCGGAGATGTGTATAAGAGACAGCCCAGGAATACATCCTTGGAA
gDNA Off-Target 6 FWD (ABE1)	TCGTCGGCAGCGTCAGATGTGTATAAGAGACAGGCCAGGAATAATGGGTTGTGA
gDNA Off-Target 6 REV (ABE1)	GTCTCGTGGGCTCGGAGATGTGTATAAGAGACAGggagtggggagggatagcat
gDNA Off-Target 7 FWD (ABE1)	TCGTCGGCAGCGTCAGATGTGTATAAGAGACAGGAATCTTGGGCACCATGGAA
gDNA Off-Target 7 REV (ABE1)	GTCTCGTGGGCTCGGAGATGTGTATAAGAGACAGTGACCAATCTCCTTGATGAGGAA
gDNA Off-Target 8 FWD (ABE1)	TCGTCGGCAGCGTCAGATGTGTATAAGAGACAGGCCCAGACCCTTCAGACAGA
gDNA Off-Target 8 REV (ABE1)	GTCTCGTGGGCTCGGAGATGTGTATAAGAGACAGGGCATTCTGATCCCAACTCCT
gDNA Off-Target 9 FWD (ABE1)	TCGTCGGCAGCGTCAGATGTGTATAAGAGACAGTGGGGCCACTCAGAGTTGAT
gDNA Off-Target 9 REV (ABE1)	GTCTCGTGGGCTCGGAGATGTGTATAAGAGACAGgcctcttcaggcaaccctct
gDNA Off-Target 10 FWD (ABE1)	TCGTCGGCAGCGTCAGATGTGTATAAGAGACAGTTCCAGCCTCTGACAATAAGCA
gDNA Off-Target 10 REV (ABE1)	GTCTCGTGGGCTCGGAGATGTGTATAAGAGACAGGGCAGGTTTGTAGCTTTCTTGC

sgRNA sequences:

Description	sgRNA Sequence
SpCas9 ABE16 target guide (ABE)	GGGAATAAATCATAGAATCC
SaCas9 ABE16 target guide (ABE)	GGACAAGGTTTGAAGGACAGG
SpCas9 YFP target guide (ABE)	GTGAACCGCATCGAGCTGAA
SaCas9 RNF2 target guide (ABE)	CATGAAAACTTAAATAGAACA
SpCas9 YFP target guide (CBE)	CCGTCCAGCTCGACCAGGAT

SpCas9 RNF2 target guide (CBE)	GTCATCTTAGTCATTACCTG
SaCas9 PCDH15 target guide (ABE)	GGTTCACCTCTCATTCAGATT
SaCas9 ABE1 target guide (ABE)	CTGAATAGCTGCAAACAAGTG
SaCas9 ABE3 target guide (ABE)	CGGTGAACTCAGAGATAGAAA
SaCas9 ABE4 target guide (ABE)	TGAAGGGAAATCTGGAGCAAA
SaCas9 ABE5 target guide (ABE/CBE)	CTCAGAAAGCTCAAAGTGTGA
SaCas9 ABE7 target guide (ABE)	AAGAATACTAAGCATAGACTC
SaCas9 ABE8 target guide (ABE)	TGGGCTTTAGGAACAGGGGTA
SaCas9 ABE9 target guide (ABE/CBE)	AACAACAAAACGGACAGTGAA
SaCas9 ABE11 target guide (ABE)	GGGAGCTAGACAAAATGGAGT
SaCas9 HEKsite4 target guide (ABE/CBE)	GGCGAGGCAGAGGGTCCAAAG
SaCas9 ABE17 target guide (ABE)	ATTTACAGCCTGGCCTTTGGGG
SaCas9 ABE18 target guide (ABE)	GGAGAGAAAGAGAAGTTGATTG
SaCas9 ABE19 target guide (ABE)	GTGTCAGGTAATGTGCTAAACA
SaCas9 ABE21 target guide (ABE)	GCTGTTGCATGAGGAAAGGGAC
SaCas9 ABE27 target guide (ABE)	GGGAGCTCAAGCCTGATTCCAA

On-Target sequence used for alignment of amplified sequences.

Description	gDNA Sequence
ABE16	CAGCAATCCAGCAACACGCGGGGAGGTGGAGAGAGGATGTTTTGCTTATCCAGAAAAGGGAGTGATTGCTTCCAGGGGCCTCAGGGGAATAAATCATAGAATCCTGGACAAGGTTTGAAGGACAGGTAGGATTTGGGTGGGTGGAGGAGGGTGCATGGGGTCAGAATTGTAACCGAAAACTCATTCCAGGTGGATAGAGC
RNF2	CCAACATACAGAAGTCAGGAATGCTTGAATATAAATTTATTATTACTCTATGTTCTATTTAAGTTTTCATGTTCTAAAAATGTATCCCAGTTTACACGTCTCATATGCCCCTTGGCAGTCATCTTAGTCATTACCTGAGGTGTTCGTTGTAACTCATATAAACTGAGTTCCCATGTTTTGCTTAATGGTTGAGTTCCGTTTGTCTGCAC
YFP (ABE)	CTTCTTCAAGTCCGCCATGCCCGAAGGCTACGTCCAGGAGCGCACCATCTTCTTCAAGGACGACGGCAACTACAAGACCCGCGCCGAGGTGAAGTTCGAGGGCGACACCCTGGTGAACCGCATCGAGCTGAAGGGCATCGACTTCAAGGAGGACGGCAACATCCTGGGGCACAAGCTGGAGTACAACTACAACAGCCACAACGTCTATATCATGGCCGACAAGCAGAAGA

	ACGGCATCAAGG
YFP (CBE)	GCGCCAAGATCCATTCGTTGGGGGATCCACCGGTCGCCACCTTGGTGAGCAAGGGCGAGGAGCTGTTCACCGGGGTGGTGCCCATCCTGGTCGAGCTGGACGGCGACGTAAACGGCCACAAGTTCAGCGTGTCCGGCGAGGGCGAGGGCGATGCCACCTACGGCAAGCTGACCCTGAAGTTCATCTGCACCACCGGCAAG
YFP (CBE-AIDx)	CGCAAATGGGCGGTAGGCGTGTACGGTGGGAGGTCTATATAAGCAGAGCTCTCTGGCTAACTAGAGAACCCACTGCTTACTGGCTTATCGAAATTAATACGACTCACTATAGGGAGACCCAAGCTGGCTAGCATGTCCGTCCTGACGCCGCTGCTGCTGCGGGGCTTGACAGGCTCGGCCCGGCGGCTCCCAGTGCCGCGCGCCAAGATCCATTCGTTGGGGGATCCACCGGTCGCCACCTTGGTGAGCAAGGGCGAGGAGCTGTTCACCGGGGTGGTGCCCATCCTGGTCGAGCTGGACGGCGACGTAAACGGCCACAAGTTCAGCGTGTCCGGCGAGGGCGAGGGCGATGCCACCTACGGCAAGCTGACCCTGAAGTTCATCTGCACCACCGGCAAGCTGCCCGTGCCCTGGCCCACCCTCGTGACCACCTTCGGCTACGGCCTGCAGTGCTTCGCCCGCTACCCCGACCACATGAAGCAGCACGACTTC
PCDH15 (ABE)	CCAAGCTAATGACCGTGCCCAAAATCTGAATGAGAGGtGAACCACCACCACCACTCTCACAGTGGATGTTCTGGATGGAGATGACTTGGGTCCAATGTTTCTTCCTTGTGTCCTTGTGCCAAACACTCGTGATTGCCGTCCACTCACTTATCAAGCTGCCATACCTGAGTTGAGAACTCCGGAAGAACTGA
ABE1 (SaCas9 site)	ACAAGACCTGGCTGAGCTAACTGTGACAGCATGTGGTAATTTTCCAGCCCGCTGGCCCTGTAAAGGAAACTGGAACACAAAGCATAGACTGCGGGGCGGGCCAGCCTGAATAGCTGCAAACAAGTGCAGAATATCTGATGATGTCATACGCACAGTTTGACAGATGGGGCTGG
ABE3 (SaCas9 site)	GTGGCAGGCAGATTATCATTCCCATCTTATAAATGAGTAAATTGAGGCTCAGAGGAGATGTGCCATGGCTTAGGTCCTGCCGACAGCCAGTGGTTAAGTCAGAACCCGACTCAGGTCAAGAAAGCAGAGACTGCCGGGGGTTGGGAAGGCGGTGAACTCAGAGATAGAAACAGGGTGGGTGGGGAAGGAGGAGCTGCTGAAGGTGAGGA
ABE4 (SaCas9 site)	GTGCTAAACAGAGAGTTACTGCTCAGACATGTAATAATAATAAATAACACATCAAATAACCATACCATTTTAAGCTGTAGTATTATGAAGGGAAATCTGGAGCAAAGAGAATAGACTGTAGGGAAACCAGTTAAGAAATAGGACATGGAGGCTAGGTGCAGTGGCTCACGCCTGTAATCGCAGCACTTTGGGAGGCTGAGGCAGGTGGATCA
ABE5 (SaCas9 site)	CATCTTACCCTCCTCAGAAGTCAAGCCTGAGCTTCCAAAAATGAAGGAACTCAGGTCCAGGGAAACTTTCTGTTTTGTCTGAGGGTGAGGGATGAGATAATGATGAGTCAGGGCTTCAGGGAAACGAACATTTACTGATTACCTAATGTGGGGGTAGATGTGGTCCCTGCTCTCAGAAAGCTCAAAGTGTGAATGAATTAAAGTCAATGTGACCAGGC
ABE7 (SaCas9 site)	GTTCCACCGCCTTGTTTACTGCTCAGTCCCCCAGAATGGCAGGCACGTAGTAGGGCTCAGTAAATATTTGCTGAATGAAGTTATGATCGAAAGAATACTAAGCATAGACTCCAGGATAAGGTAAGTAATATAGAGTTCCACAGTGGGCAGGTCTATGCAAACCTACCCCAAAGTCCGAGGAAGCTGAGAGGGCGAAGAAAGAGGCTGAG
ABE8 (SaCas9 site)	CATACTTAAAGTAAAGCAGAAGGAATAACAGTGCCCCAAATTATTCACTACCCCTGTTCCTAAAGCCCACCATCTTTTGTACACTCAGAGTAAACAAAGCATAGACTGAGGGGTACAATCCTACTCTAGTCCCTTTCCTCATGCAACAGCTATGAAATAGCTGGTTAGTACTGACA

	GCAATTCTTGGCCACAAACAGAACCAGTTCTATAGTGGAATGAAACTTC
ABE9 (SaCas9 site)	ATCATAGCGAGACCCTGTCTCAGCAACAACAACAAAACGGACAGTGAAGAGAGTATTTGAAGGAGAGGGGAACAGGCAGCGTATTGCTTATTGCTGAGGGGCAAAGTGAAGACCAAGGATAGACTGCTGGGCTTGACAGCATGGAGGGTGCTGGGGGCCTAGGCAAGTGCAATATTCATGTGGTGTCATTGGGCCAAAGGTGTCATTGGAGAGTACTC
ABE11 (SaCas9 site)	GAGGACTACTGCATCTTATTGCCTGCAAGAAAATAATTTTAATACTAAATTCTGGAAATGACATGCTACTCACTTGCTTCCTCCATGGAAAAATTATATCTTGAGATACAAGGACAGGCAGCATAGACTGTGGGTATAGAATGTGGCTGGGGCTGCATGAAAAGAAAAGTTATTGGGAGCTAGACAAAATGGAGTTCGAGTTCTTGTCTTTGGCAAAATTGCTGTCTCTTTCTAGGCCTTCATTTCC
HEKsite4 (SaCas9 site)	TTCAAGATGGCTGACAAAGGCCGGGCTGGGTGGAAGGAAGGGAGGAAGGGCGAGGCAGAGGGTCCAAAGCAGGATGACAGGCAGGGGCACCGCGGCGCCCCGGTGGCACTGCGGCTGGAGGTGGGGGTTAAAGCGGAGACTCTGGTGCTGTGTGACTACAGTGGGGGCCCTGCCCTCTCTGAGCCCCCGCCTCCAGGCCTGTGTGTGTGTCTCCGTTCGGGTTGAAAGGA
ABE17 (SaCas9 site)	GGACATTTCCACCGCAAAATGGCCCCTCTGGTGGTGGCCCCTTCCTGCAGCGCCGGCTCACCTCACGGCCCCGCCCTTCCCCTGCCAGCCTAGCGTTGACCCGACCCCAAAGGCCAGGCTGTAAATGTCACCGGGAGGATTGGGTGTCTGGGCGCCTCGGGGAACCTGCCCTTCTCCCCATTCCGTCTTCCGGAAACCAGATCTCCCACCGCACCCTGGTCTGAGGTTAAATATAGCTGCTGACCTTTCTGTA
ABE18 (SaCas9 site)	TGTGTATGGTGAGAGGTAGGGATCTAGTTTCATTCTTCTACATGGTTGTAAACTGTAGTCACCCTACTGATCTATCAAACACTGGGTCATACTTCTTCTATTAAACTGTATGTTTGTACCCATCAATCAACTTCTCTTTCTCTCCCCCACCCCTCTACTCTTCCTGACCTCTAGTAACCAGCAGTCTATTCTCTATCTTCATGAGATCCACTTTTTTAGTTTCCACATGTGAGTGAGAACATTTGATACTTGTTTTTTTTCACATTTGTTATACTCCTGGGCTGAAGTG
ABE19 (SaCas9 site)	CTGCACCTAGCCTCCATGTCCTATTTCTTAACTGGTTTCCCTACAGTCTATTCTCTTTGCTCCAGATTTCCCTTCATAATACTACAGCTTAAAATGGTATGGTTATTTGATGTGTTATTTATTATTATTACATGTCTGAGCAGTAACTCTCTGTTTAGCACATTACCTGACACTAAATAAATGTTTAGCAATTATAGTAATTATATGTGGAATAAATGAAAGAAAAGCAGCTGAACACTTAAGGAGATAAGAAACCATGTCTCTGGATGCCACAGC
ABE21 (SaCas9 site)	CCCTGTTCCTAAAGCCCACCATCTTTTGTACACTCAGAGTAAACAAAGCATAGACTGAGGGGTACAATCCTACTCTAGTCCCTTTCCTCATGCAACAGCTATGAAATAGCTGGTTAGTACTGACAGCAATTCTTGGCCACAAACAGAACCAGT
ABE27 (SaCas9 site)	CGCGGGCTGAAGTAGATCAAGTCCCCAGGGAGCTCAAGCCTGATTCCAAGGAGATTGCCAATATTTTAGGAGGGAGTAAACATTTTCTGAAGTTTTTTGTTGTCTAAAGCCAAAAAATGCACTTGCGCCTCCTGAGAGGACAAAGAGGAAGAGAGACGGGGAGAGGGGACAAAGGAGCAGAGAC

Off-Target sequence used for alignment of amplified sequences.

Description	gDNA / cDNA Sequence
DNAJB (mRNA Off-Target Site 1)	GCGCTACCACCCGGACAAGAACAAGGAGCCCGGCGCCGAGGAGAAGTTCAAGGAGATCGCTGAGGCCTACGACGTGCTCAGCGACCCGCGCAAGCGCGAGATCTTCGACCGCTACGGGGAGGAAGGCCTAAAGGGGAGTGGCCCCAGTGGCGGTAGCGGCGGTGGTGCCAATGGTACCTCTTTCAGCTACACATTCCATGGAGACCCTCATGCCATG
MTA2 (mRNA Off-Target Site 2)	GGGATTCGTTCAAGCTCACAGCCAGCAGCCAAGCGTCAGAAACTAAACCCAGCTGATGCCCCCAATCCTGTGGTGTTTGTGGCCACAAAGGATACCAGGGCCCTACGGAAGGCTCTGACCCATCTGGAAATGCGGCGAGCTGCTCGCCGACCCAACTTGCCCCTGAAGGTGAAGCCAACGCTGATTGCAGTGCGGCCCCCTGTCCCTCTACCTGCACCCTCACATC
PTBP2 (mRNA Off-Target Site 3)	AGATTTTGGTAATTCCCCATTGCATCGTTTTAAGAAACCTGGATCCAAAAATTTTCAAAACATTTTTCCTCCTTCTGCCACCCTTCACCTATCTAATATCCCTCCATCAGTAGCAGAAGAGGATCTACGAACACTGTTCGCTAACACTGGGGGCACTGTGAAAGCATTTAAGTTTTTTCAAAGAGATCACAAAATGGCTCTTCTTCAGATGGCAACAGTGGAAGAAGCTATTCAGG
SAP30BP (mRNA Off-Target Site 4)	CAGAACCCCCTGGCAGATGTTCAAATCACTTGCAAGACAAGATCCAGAAGCTTTATGAACGAAAGATAAAGGAGGGAATGGATATGAACTACATTATCCAAAGGAAGAAAGAATTTCGGAACCCTAGCATCTACGAGAAGCTGATCCAGTTCTGTGCCATTGACGAGCTTGGCACCAACTACCCAAAGGATATGTTTGATCCCCATGGCTGGTCTGAGGA
LCMT1 (mRNA Off-Target Site 5)	ATTGCCAACACTCCTGATAGCTGAATGTGTGCTGGTTTACATGACTCCAGAGCAGTCCGCAAACCTCCTGAAGTGGGCAGCCAACAGTTTTGAGAGAGCCATGTTCATAAACTACGAACAGGTGAACATGGGTGATCGGTTTGGGCAGATCATGATTGAAAACCTGCGGAGACGCCAGTGTGACCTGGCGGGAGTGGAGACCTGCAAGTC
SCAP (mRNA Off-Target Site 6)	AGCAGACCTGAACAAGCGACTGCCCCCTGAGGCCTGCCTGCCCTCAGCCAAGCCAGTGGGGCAGCCAACGCGCTACGAGCGGCAGCTGGCTGTGAGGCCGTCCACACCCCACACCATCACGTTGCAGCCGTCTTCCTTCCGAAACCTGCGGCTCCCCAAGAGGCTGCGTGTTGTCTACTTC
gDNA ABE11 Off-Target Site 1	CAGGCTGCCAACCATACTCAAGACTAATGCAAAATTTAAGCAGGGTTGAGTTTTCTGGCTGAAAATATAATAACATAGGGTCAGGATGATATAAAAGCTTAGACTTCTGCAAACGCACTCCATTTTGTCTATAACCTTTCAAGATTGCTTTAGTTATCTATTACCATGTAACAAACCACTAACTCTTAGTGATAAAACAAAATAACTATTTTTAATCTCTCATTTTCCTGTGGTTTGACTTGAATCAGC
gDNA ABE11 Off-Target Site 2	CCTGCTTACAGGACTTGTGTCCAAAGGCAGACTCCATTTTGTCTCTTACCTGCATCCCCCAAAATGATGATGACACCATATACTCTGTGTTAACCTGATGAGCCACTCAGAGCCCCACATGAGGTCACAGCCTTATGTTCTCCAGGACCTGGCCAATCAATCACAGGTTCATATAGTCCTCTGAGTCATTAAGTCACCTCCACTGCCCCATTTTCAG
gDNA ABE11 Off-Target Site 3	ACCTCTCCCACTCCAGGTCATTTCACATCAACTCTTTCTGCCTCCCTCTTGTAAGTGTGATACCCTACCTTGTTTTAACCTGAATTGACTGTCCCTTAGCTGAGAGAGCCAGACACACTCCATTTTGGCTCCTTCACTTGCAGCCCCTTACCCACCCCCCTTCCTCAAGGACTTAACTTGTGCAAGCTGACTCCCAGCACATCAAAGAATGCAATTAATGGATAAGATACTGTGGCAAGCTATATCCGCA

gDNA ABE11 Off-Target Site 4	ACCTCTTCGAGTGGGCAGTGAGTTCAGGCAGGGGTCACTCACCAGGCGCCCTGGCCCCTGTCCAGCTGCACAGGCCCTGGAGGGAACCCAGAGAAAACGGAGTCAGCCTCTCAACCACTCCTCCGCCCAGTGAGGCCGAGGGGCTGGGAGGAGCGGCTGGTCCCCGCCCCATGAGTCACCCTTGCATGGGAAGGAGGGGCCGGTGTTAGGCTGGGCTGTCACGACCTCGACCGTCTTT
gDNA ABE11 Off-Target Site 5	CTGCAAGCTCAAAACCCTGATATGTAAATCAGAATGGCCAATTTGTTAACCTACAGCAGCACATAAAAACATCAAATGTACAATGTATCGTTTACATCTGATCAGTATTTATCTCCTTATAGGTAAGCTACATAAAAAGGAGTTTGCTTGGTGAGCAATGGTGGAGTGAGGCTCATGATTTGAGTATTTCAAGATGTCAACTAAGGGCACCTCAACCAAATGCACCTCATGCC
gDNA ABE11 Off-Target Site 6	TCTTGTGCCACTGTGCATGATTACAGAATTACACAAAACCTCCACAAAGGATTACAATACAAACACCTTTATATCTGCCAGATATATAATATTTAGGAAGGATTTGTATAGGGTGCTATGAGTGGATCAATGATTGCTGTAGAAATGAAGTTTATGGGGAGCTAGATAATGCGGAGTTAGCTTAGCTAATGGGAATGTGTAGATAGGATTCCAGGAAGGGGAAGCA
gDNA ABE11 Off-Target Site 7	TTGTTCTGCACTTTTTGTTGGAATAAAGAATTCCAAGGCTAACTACATATAGCCTAGCTCCCTCATTTTACAGATGAGCAAAAAGAGACCAGAGAAAATAAGTCCGATCAACCAGATTAAATAATACAGTAAATGAAAACTTGGATTAGAATCTAGTTTTCCTAATTATCTAGTACTCTATGCCCTGCACAATACTCCCT
gDNA ABE11 Off-Target Site 8	TTCATGAGGAATTTGCAACACAGTGTGTTTATTACATAAGTTAAGCTTTTTCCTACTTAAGGCATTTAAAAACACACTACATCTGATCTAGCTCCCCTTTAAACTCTTCCTACATCCTTTTTCTTTACCCTGTTCACATAAATACATTGCTACCCATTTCCCAACGGCCTTAATCTGATACGACATTCTGCCCACAAGAG
gDNA ABE8 Off-Target Site 1	CCTGGGAGTTCCTCCAGACCTGCTGCCGGAGCCAAGCTGGGAACCCACTGCCCTTTCCTTGCCAAGCCAAGGCTGTATGCTGGGCATTGTCGGGGAGGTAGAGGCACCCTATGGGCCAGGTGGGGACACTCTAAGCCATACCCCTTTCCCTACAGGCCTGGCTACAGCAATATGGCTACCTGCCTCCCGGGGACCTACGTACCCACACACAGCGCTCAC
gDNA ABE8 Off-Target Site 2	ACTTGGGAAAAGTAAACACTCAAAAATAATCTTAGGAAGTTTGAAAATATTTTCATGGTAGTAATCTGATCACTAGGGTTACATTCTTTGATATCCTACCTTTAGCATGTCATAGGTTTTAGGAACAGGGGAACGGCCTCCAACAAAATCATTTTCAACCAAGTGTAGGTTGTAGACATACTCAGGAACACTGCTGGTTCGGTGAAGA
gDNA ABE8 Off-Target Site 3	AAGCTGCGGTGTTGAATGGTGTGTCTTAGACCCGGGTGCCTAGTGTGGCTCGGTGCCTTACAATGAATTTGGGGAAAGAGTTGTCAAACGAAACCCATGTTTCTAATGACCAGCAGGTAAGTAGTTCCGGCTTTTTCTTTTCATTTAATCAGTGGTAGGAAAGCGTGGCACCCTGGCTATCCTGTTTTAGCGAGTGGCAGCTGTG
gDNA ABE8 Off-Target Site 4	CGGAAATCTTCACCGAACCAGCAGTGTTCCTGAGTATGTCTACAACCTACACTTGGTTGAAAATGATTTTGTTGGAGGCCGTTCCCCTGTTCCTAAAACCTATTACATGCTAAAGGCTGGCACAACTGCCACTTATGAAGGTCGCTGGGGAAGAGGAACAGCACAGTACAGCTCCCAGAAGTCCGTGGAAGAAAGGTATTTGAGGCATCCTCTGAGGAGACTGGAGATTTCTCCTGACAGCAGCCC
gDNA ABE8 Off-Target Site 5	TTGGCATTTCCAAATACCCTCTTTTTACATCTAATTTCAATAAATTCCGTACTCCATTAAAAGAGATTAATGTATGAGGCCTTAGGAAAAGGGGTAGGGCTTAAACATCTCTACATATACCTGAATAACTTACTAGAGTGCCTTATACAAAGAAGTTCAAGAAATGTTAGTCTCCTCCCTTCTCAGTAGAAAAGCAAAATCCTATTTACTACTAGAAATGGGCTTACTTTCCTGGTCCAGCATTCA

gDNA ABE8 Off-Target Site 6	CTTCCTCATGCTGCCCTTCTATAGTCAAACCCTACCCCTGCTCTTAAACCCTGGTCATCACTGATCTCTTATCCTTCTCCATAGTTTTGTCTTTTCCAGAATATCATAGAAATACAATCATGCAGAATGTAAACTTTTAAGATTGTCTTCTTTCACTCAGTATCATGCTTTTGAGATTTATTCATGTTCTGTGTATAAATCATTCATTGTTTATATTGCTGCTGAGCA
gDNA ABE8 Off-Target Site 7	TGTCCAATGACAGGAGCATGACAACATTCCACCTATTCATTCACTCACTCATCCTGCATTGAGTGAGTGACTGCTGTATGCCAGTGCCGACATGCAAGACTTCTCAAAATTTCTTTCAGTTCTAACGTTCTCAGGTTTTAGCAAGAGGGGTATTGTTTTGCCCCAAAACAGGCCATCTCTCACATTTTCAGACTGGTATCCATTTGTGGCTTTTGTGC
gDNA ABE8 Off-Target Site 8	GCCCCTGAGCCATTAAAACCCAGACTAGCCCAGCACTGGTGCGTTCTTCTCTGTCCCCGTCCTGAACCTGCCAGGCTTTCCCTGAGGTCCCCAGGCACCAGGGGATTATTGTCAGGCCCCTCACTGTCGTCCTGGGCCCTCGGAATCATGCCTCTGTGCCCAGAGTGGTGCTTTGGAAGAGGGGTATGGCCTTGTCACTGGGGTCCTTCATGGCCCTCACCTGTTT
gDNA ABE8 Off-Target Site 9	CAGAGACTCGGGGGACTGAAGTCTCTGACATGAAGCGCTTCCTGCAGGGTATAAGCATTTGGAACACGGGTAAGGCCTATGGGCTAAAATCTCCAGCCAGCAGCGGTTAGAAATTCAATGTTGTCCCCAAGCACTTTAGATCTCCTGAGTGTTTTGAATTCCAGTGGGAAGGGAGTTCTGATAAAAGGCAGCTGAGTTTGGAAGGATTGTAGCCTCAGGATGGCTCCTCTGCCTTTTGACCCATG
gDNA ABE8 Off-Target Site 10	AGGCCCATATGTGCTCCAGAGTTGTTCTCCAGTAAGGAGAGCATCACCGTGCTTCAGAAACTGGGGTAGAGCTTGCACTTTATGTAGTTATTTAATTACTCCAACACCATATAAAGCTCTCTTTCTCTGATGAATTTCCTTTGTACCCTTGTCAAAAATGAATTGACTATGTATATGCAGGGCTATTTCTAAACTCTGTT
gDNA ABE1 Off-Target Site 1	CCCTGGGCTATTTTTGTCCACTGTACCTACTGTTGTTGGGACAGTCCTCATTCCAGCTTAACAGTGGGAAAACTAAATTTAGCCCAGTCCAGGAACTGGACAGACACCACTTGTTTGGCCCTTTTCAGTTAAAACTATGGGTCCAGTACAGTTCAAATTATAGATATTAGGGCTTCTTCCCCTTGGGAAACTTGCTTTACAGCTGTGGCTGAGAATATGGCCA
gDNA ABE1 Off-Target Site 2	AGTCCCGACAGGCTTTCCATTTGTTTCCAATGGCTTTATTCCACATGACTTTAGTAAACCGTACTCAATCAATTTCTGACCTTGGTACTTTGAAGCAACACTTGTATACAGATATTAAGGGAAGAGGGAAGTTAAGGGAGGAAAATCGAAGGTCAAACTGTGTGTCAAACAAGGTGCTGGGTCCTAATTTTATTTTTAGTTGCCTCTTGAATCGCTCAAGCCCATCT
gDNA ABE1 Off-Target Site 3	TGCCCTTATGAAGAGAAGGAGGACAAGCCTTATTTCTCATCAATCTGCAGACTGATCCTAGTGACAATCTGCTGTCTGTCCTAGAGACTCCAGAGTAGCTCCAAACCAGTGCAGCCTTGGATGAGAGTAGACCAAGCCCTCCCTGTCTCAGAATCTCTCTAGTCCTTAAGGAACACTATGGATTGGCTTAGGGACCCAA
gDNA ABE1 Off-Target Site 4	TGAAAGCTAGGCATGGGTGAAAACTACACTTCTTTACATCTTTTCAGGACTTAGGTATCTTTTATGTTGGCTGACTTTTATGTGGAGCATCAACCCTGTGTGAGGTGCTATGGAAGGTGGGGATAGTGGGAGAAGCAATGTAGGATAATGGCTAAGAACACAGACTCCAGAGCCGACTGTGTGGGTCTCAGCTGCACCTTCACTAAATCTTTCTGGGACCTTGGGAATG
gDNA ABE1 Off-Target Site 5	TGAGCATTTCTCTCAGGTGTCCATGAATTTAATGAAAATTACTTAATTTTTATTATAATTTCAATAATAATATAAGAAATATCTTATTTGTTATGAAGATAAAACTTCTCTTTTGCCCTGAAAACCTGCAAACAAGGGGAGTTTCACGGAATAAGAGATAAATTTTTTTTTATTAGTGTTTTTTGAAATAACTTCCAAGGATGTATTCCTGGG
gDNA ABE1 Off-Target Site 6	GCCAGGAATAATGGGTTGTGAACCCTAACATTCCTGAGAAGTTAGACAGCACCTGTGTGCAGTTATTCAAGATATCTTTTTTTTTAATTATTTTACTTTAAGTTTTAGGGTACATGTGCACAACGTGCAGGTTTGTTACATATGTATACATGTGACATGTTGGTGTGCTGCACCC

	ATTAACTCGTCATTTACATTAGGTATATCTCCTAATGCTATCCCTCCCCACTCC
gDNA ABE1 Off-Target Site 7	GAATCTTGGGCACCATGGAAACTGATATCCTTGAACACGCAGAAACTTGGGTTCAAGTTTTGGCAAATCTCTACTGATTAGCAGCAAACAAGTTGTGTTTCTCATGTCACTTTTGTTGTTTATTGTTTATACAATAGTCTGCCTTTTCTCTTCTCTCTCCTTTTTCCCTCCTATGGGTGGCTGCTTTCCTCATCAAGGAGATTGGTCA
gDNA ABE1 Off-Target Site 8	GCCCAGACCCTTCAGACAGAACTTCTTTGGCAGTATTTAAAAGCCATTTGAATGATGCCAGGACTAGCTAAACTTCGTTTCTGAACAGCGGCAAACACCTGGTGTTTTAAAAGTAACAGTCTAATCACATAACTAGGGCTGAGTGATCTGCAAGCTGGCCTTGACTTGTCTAGAAAGCACAATAGAAATAGGAGTTGGGATCAGAATGCC
gDNA ABE1 Off-Target Site 9	TGGGGCCACTCAGAGTTGATGTACACACTAGTGTCTAATTTCTGCCTGAGAATAGAGTAAGACATAGTCTGAGTAGCTGCAGACAAAAGAGGCCTAGGCATGGCTACACTAGGAGGACTGTGCACCCAACATTCACTAGGGGGAAAGGAAGCATGACCATTTCTCATGGACCAGTGATAGACCAGGTATAAGATAGAGCTGTGTATGAGCCAGAGGGTTGCCTGAAGAGGC
gDNA ABE1 Off-Target Site 10	TTCCAGCCTCTGACAATAAGCACTTTTTCCTCTATGACATTAATTTACTCCTTAAAGTTTTCTCTTCAGCTATAATCTTAAGGTAAATGGGAAATAGACATAAGTGCATTTAAATTTATAATGCACTGATTCTCTTGTGAAGGTAGTCTTGTAATGAGGAAGGAATTCAAAACAGAACTTGTTTGCAGCTCTGCAAATGTGCAAGAAAGCTACAAACCTGCC

Supplementary Table 3. SpCas9 permutants of ABEmax are compared for their on-target editing at the *ABE16* and *YFP* loci. The p-value, t-value, degrees of freedom (df), the mean difference and 95% confidence interval (CI) of the mean difference are shown for circularly permuted (CP1029-C, CP1029-N/C, CP1029-N variations of ABEmax) and intradomain base editors (ID1058). Welch Two-sample, two-sided t-tests were performed for all samples using R (v. 1.2.5019).

Comparison	Site	Mean difference	95% CI of mean difference	df	t value	p value
ID1058-ABEmax vs. ABEmax	*ABE16*	0.170201	-0.3459208 to 0.6863229	2.4951	1.1805	0.3381
ID1058-ABEmax vs. ABEmax	*YFP*	-0.2910642	-0.54542781 to -0.03670052	2.1492	-4.61	0.0383
CP1029-N/C ABEmax vs. ABEmax	*ABE16*	0.2557402	-0.3172852 to 0.8287657	2.0594	1.8681	0.199
CP1029-N/C ABEmax vs. ABEmax	*YFP*	-0.35545289	-0.61617168 to -0.09473409	2.0675	-5.6862	0.0274
CP1029-N ABEmax vs. ABEmax	*ABE16*	0.2233756	-0.2596459 to 0.7063972	3.1518	1.4324	0.2433
CP1029-N ABEmax vs. ABEmax	*YFP*	-0.36247793	-0.5965396 to -0.1284163	2.5304	-5.4885	0.01831
CP1029-C ABEmax vs. ABEmax	*ABE16*	0.3054103	-0.2756743 to 0.8864950	2.0177	2.2425	0.153
CP1029-C ABEmax vs. ABEmax	*YFP*	-0.36354865	-0.62726903 to -0.09982826	2.0327	-5.8407	0.02704

Supplementary Table 4. SpCas9 permutants of ABEmax are compared for their off-target editing at several promiscuous RNA transcripts. The p-value, t-value, degrees of freedom (df), the mean difference and 95% confidence interval (CI) of the mean difference are shown for circularly permuted (CP1029-C, CP1029-N/C, CP1029-N variations of ABEmax) and intradomain base editors (ID1058). Welch Two-sample, two-sided t-tests were performed for all samples using R (v. 1.2.5019).

Comparison	Site	Mean difference	95% CI of mean difference	df	t value	p value
ABEmax vs. CP1029-C ABEmax	*DNAJB*	-0.3393451	-0.4611651 to -0.2175250	13.614	-5.9905	3.72E-05
ABEmax vs. CP1029-C ABEmax	*MTA2*	0.0297972	-0.02156468 to 0.08115909	15.616	1.2323	0.2361
ABEmax vs. CP1029-C ABEmax	*PTBP2*	-0.0327717	-0.11106515 to 0.04552172	14.977	-0.89229	0.3863
ABEmax vs. CP1029-C ABEmax	*SAP30BP*	0.0058073	-0.03618323 to 0.04779776	14.353	0.29594	0.7715
ABEmax vs. CP1029-C ABEmax	*LCMT1*	-0.0197466	-0.08451419 to 0.04502095	14.424	-0.65211	0.5246
ABEmax vs. CP1029-C ABEmax	*SCAP*	0.0890025	0.03068875 to 0.14731614	13.336	3.2889	0.005696
ABEmax vs. CP1029-N/C ABEmax	*DNAJB*	-0.3143563	-0.4639470 to -0.1647655	11.741	-4.5899	0.000657
ABEmax vs. CP1029-N/C ABEmax	*MTA2*	0.0218489	-0.02760433 to 0.07130205	15.014	0.94161	0.3613
ABEmax vs. CP1029-N/C ABEmax	*PTBP2*	-0.0633728	-0.130504238 to 0.003758504	15.999	-2.0012	0.06263
ABEmax vs. CP1029-N/C ABEmax	*SAP30BP*	-0.0093725	-0.05269451 to 0.03394937	15.082	-0.46091	0.6514
ABEmax vs. CP1029-N/C ABEmax	*LCMT1*	-0.0642578	-0.120681366 to -0.007834312	15.73	-2.4176	0.02815
ABEmax vs. CP1029-N/C ABEmax	*SCAP*	0.0510479	-0.00552741 to 0.10762317	12.085	1.9644	0.0729
ABEmax vs CP1029-N ABEmax	*DNAJB*	-0.1968339	-0.41756098 to 0.02389321	9.6785	-1.9959	0.07483
ABEmax vs CP1029-N ABEmax	*MTA2*	0.0416136	-0.00995801 to 0.09318521	15.662	1.7136	0.1063
ABEmax vs CP1029-N ABEmax	*PTBP2*	0.025529	-0.05922721 to 0.11028514	14.137	0.64543	0.529
ABEmax vs CP1029-N ABEmax	*SAP30BP*	0.02741663	-0.02043076 to 0.07526402	15.998	1.2147	0.2421
ABEmax vs CP1029-N ABEmax	*LCMT1*	-0.0257277	-0.08072827 to 0.02927279	15.88	-0.99224	0.336
ABEmax vs CP1029-N ABEmax	*SCAP*	0.0821117	0.01039632 to 0.15382707	15.915	2.4283	0.02741
ABEmax vs ID1058-ABEmax	*DNAJB*	-0.3002663	-0.4423766 to -0.1581561	12.152	-4.5972	0.000594
ABEmax vs ID1058-ABEmax	*MTA2*	0.050258	-0.0009715148 to 0.1014873325	15.585	2.0842	0.05398
ABEmax vs ID1058-ABEmax	*PTBP2*	0.0148402	-0.05359970 to 0.08328008	15.97	0.45974	0.6519
ABEmax vs ID1058-ABEmax	*SAP30BP*	0.02419198	-0.01810152 to 0.06648549	14.544	1.2225	0.2409
ABEmax vs ID1058-ABEmax	*LCMT1*	0.0253978	-0.02749142 0.07828701	15.997	1.018	0.3238
ABEmax vs ID1058-ABEmax	*SCAP*	0.1188224	0.06039193 0.17725277	13.409	4.3797	0.000693

Supplementary Table 5. On-target gDNA editing efficiencies of the microABE-I744 and the Sa-miniABEmax(V82G). The p-values, t-values, df, mean difference, and 95% CI of mean difference are shown. Welch Two-sample, two-sided t-tests were performed for all samples using R (v. 1.2.5019).

Comparison	Site	Mean difference	95% CI of mean difference	df	t value	p value
microABE-I744 vs. Sa-miniABEmax(V82G)	*ABE16*	0.104372335	0.09841275 to 0.11033182	3.9995	48.628	1.07E-06
microABE-I744 vs. Sa-miniABEmax(V82G)	*PCDH15*	0.0023654069	0.000948219 to 0.003782595	3.1719	5.1526	0.01234

Supplementary Table 6. Off-target gDNA editing efficiencies of the microABE-I744 and the Sa-miniABEmax(V82G). The p-values, t-values, df, mean difference, and 95% CI of mean difference are shown. Welch Two-sample, two-sided t-tests were performed for all samples using R (v. 1.2.5019).

**Comparison**	**Site**	**Mean difference**	**95% CI of mean difference**	**df**	**t value**	**p value**
microABE-I744 vs. Sa-miniABEmax(V82G)	*DNAJB*	-0.02849716	-0.03680003 to -0.02019428	13.259	-7.4001	4.63E-06
microABE-I744 vs. Sa-miniABEmax(V82G)	*MTA2*	-0.00869564	-0.012389632 to -0.005001656	14.614	-5.029	0.000161
microABE-I744 vs. Sa-miniABEmax(V82G)	*PTBP2*	0.00123583	-0.02892973 to 0.03140139	10.108	0.09115	0.9292
microABE-I744 vs. Sa-miniABEmax(V82G)	*SAP30BP*	-0.005169157	-0.007055860 to -0.003282453	14.007	-5.876	4.02E-05
microABE-I744 vs. Sa-miniABEmax(V82G)	*LCMT1*	-0.00095559	-0.004171663 to 0.002260497	15.995	-0.6299	0.5377
microABE-I744 vs. Sa-miniABEmax(V82G)	*SCAP*	0.003247691	-0.007558782 to 0.014054164	8.1066	0.69145	0.5086
G129 vs. microABE-I744	*DNAJB*	0.02022031	0.01493234 to 0.02550827	14.498	8.175	8.35E-07
G129 vs. microABE-I744	*MTA2*	0.01617262	0.01203730 to 0.02030795	13.416	8.4223	1.02E-06
G129 vs. microABE-I744	*PTBP2*	0.01171024	-0.02287688 to 0.04629737	15.208	0.72079	0.482
G129 vs. microABE-I744	*SAP30BP*	0.003052556	0.001232690 to 0.004872421	14.395	3.5883	0.002851
G129 vs. microABE-I744	*LCMT1*	0.0191464	0.01645462 to 0.02183818	12.125	15.48	2.37E-09
G129 vs. microABE-I744	*SCAP*	0.004979992	-0.006018323 to 0.015978308	9.5445	1.0155	0.3349

Supplementary Data File 1.

Genomic DNA off-target analysis based on sequence homology of sgRNA targeting *ABE1, ABE8*, and *ABE11*. The sgRNA for the top three edited genomic sites were selected and each of the three independent technical replicates were sequenced using parallel mock samples as a negative control (n=3). Data are presented as the sum of reads for each aligned sequence across the three replicates.

Supplementary Data File 2.

Amino acid sequence of microABE I744, SaCas9-miniABEmax (V82G), SaCas9-ABEmax, microAIDx I744.

Supplementary Figure 1.

Surface render of SpCas9n with key residues for permutant variants highlighted. *S. pyogenes* Cas9 (purple PDB: 4OO8) complexed with target DNA (red) and gRNA (orange) is displayed. Key residues for circular permutation (1010, magenta; 1029, cyan; 1058, green) or intradomain insertion (202, yellow; 208, navy; 468, black; 1058, green) of the hAIDx protein domain are highlighted. Numbering is considered from the starting methionine at position 1. All graphical representations generated with PyMol (Methods). BH, Bridge helix.

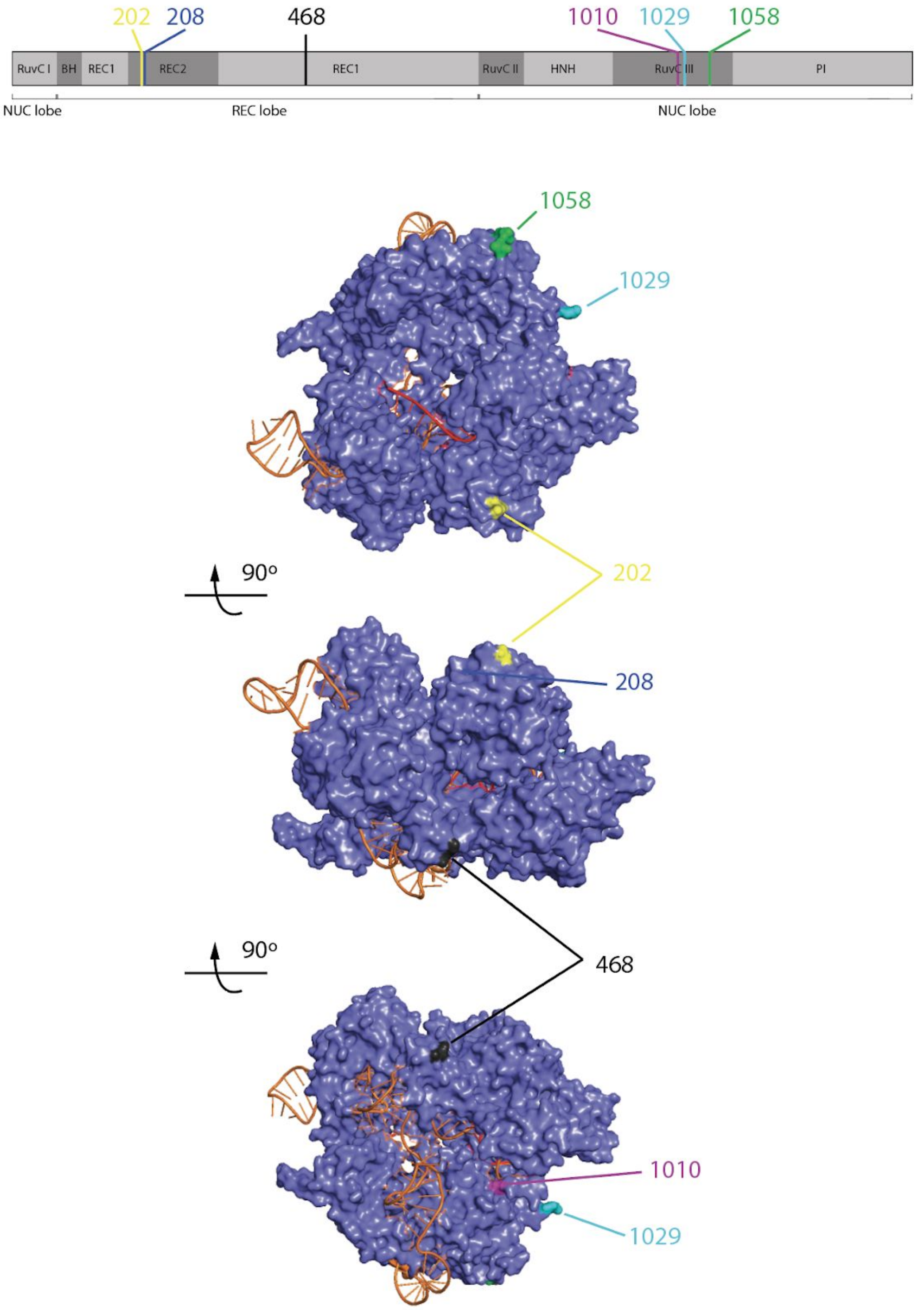

Supplementary Figure 2.

Cytosine base editors (CBEs) are shown for BE3, TAM-AIDx (as CP and ID constructs), and Target-AID (C-terminal linked), which have a C-terminal linked uracil glycosylase inhibitor. The antisense strand is shown; cytosine-to-thymine transitions are represented as guanine-to-adenine transitions

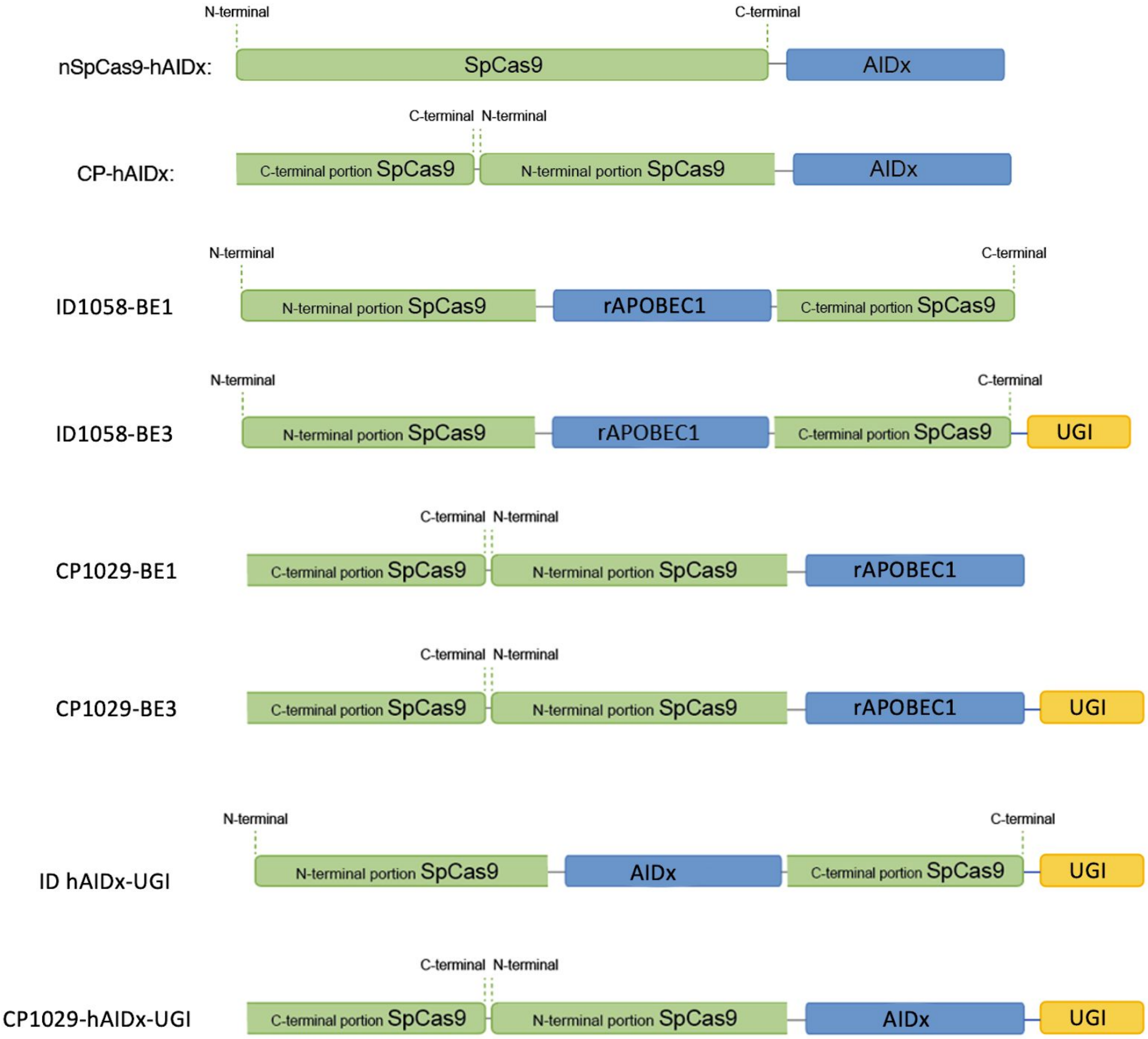

Supplementary Figure 3.

hAIDx was either circularly permuted (CP) or inserted as an intradomain construct (ID) into SpCas9. Raw data points are shown for the on-target editing efficiency for cytosine substitutions at the highest-edited position in the original TAM-AIDx construct against an sgRNA targeting the *YFP* locus.

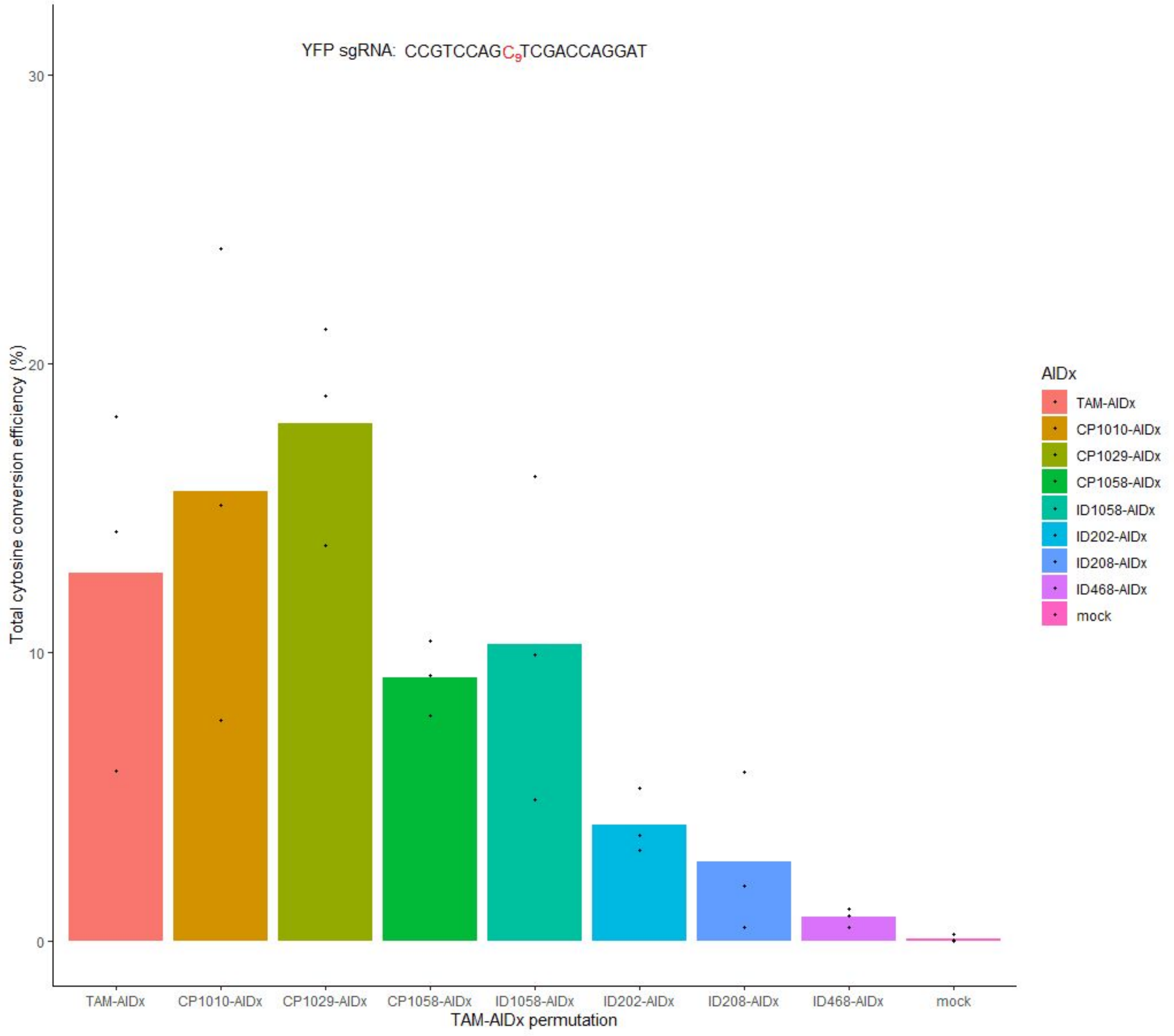

Supplementary Figure 4.

Example *YFP* site allele tables from genomic DNA isolated from HEK293A-YFP cells after editing with nSpCas9, where the hAIDx has been linked to the C-terminal, inserted in the PI domain (at residue 1058) or linked to a circularly permuted nSpCas9 (again at residue 1058). Each of these constructs had no UGI. Samples were sequenced on an Illumina MiSeq and analyzed using CRISPResso2. The reference sequence and gRNA are displayed at the top, with the frequencies and corresponding sequencing read counts being shown for each allele.

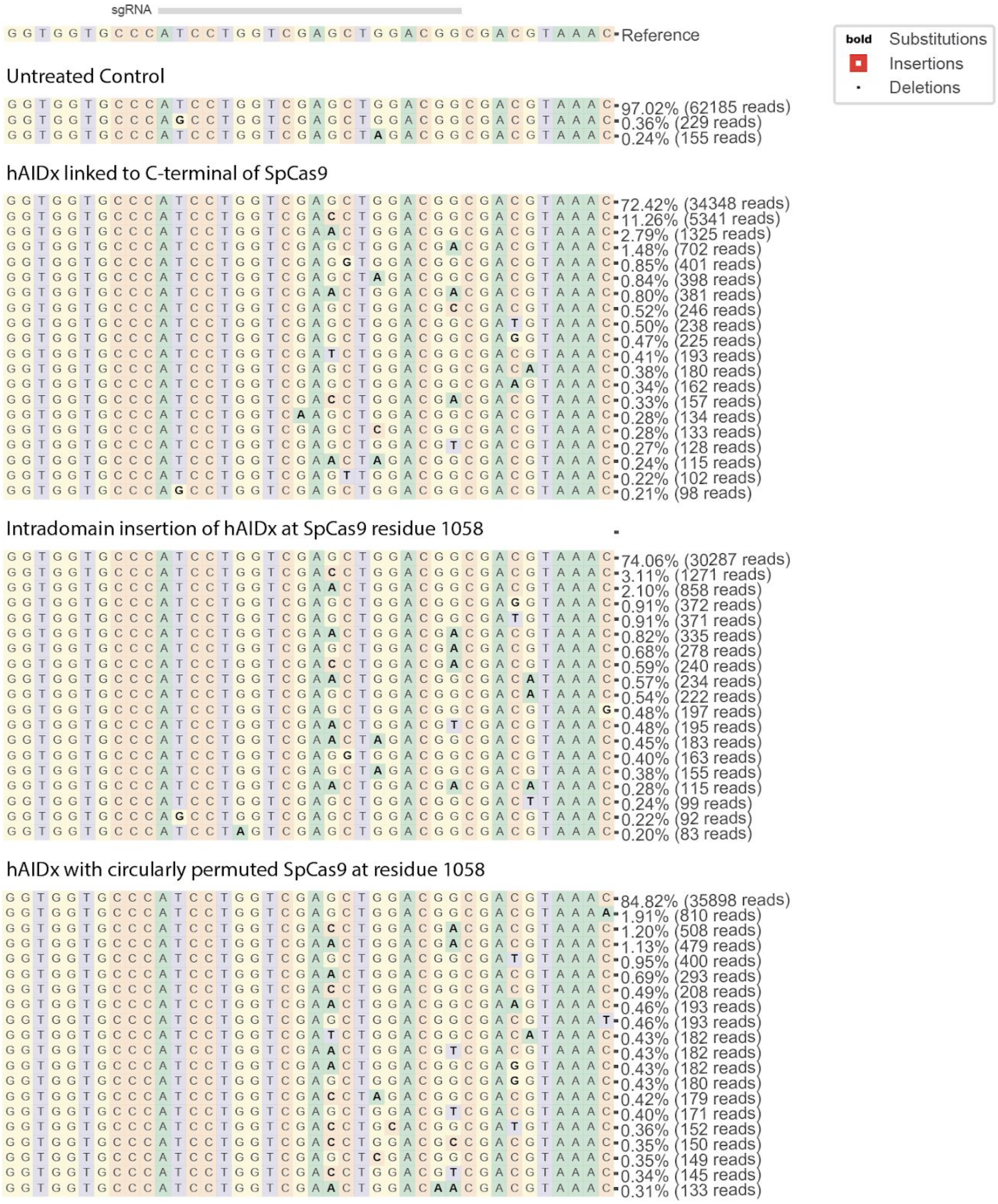

Supplementary Figure 5.

Direct comparison of various permutations of cytosine base editors (CBEs) for on-target activity across an sgRNA. CBEs permutants were compared in a head-to-head comparison targeting the *YFP* locus. Total substitution is shown for each CBE construct. Individual data points are shown for each construct (n=3 technical replicates performed on independent days).

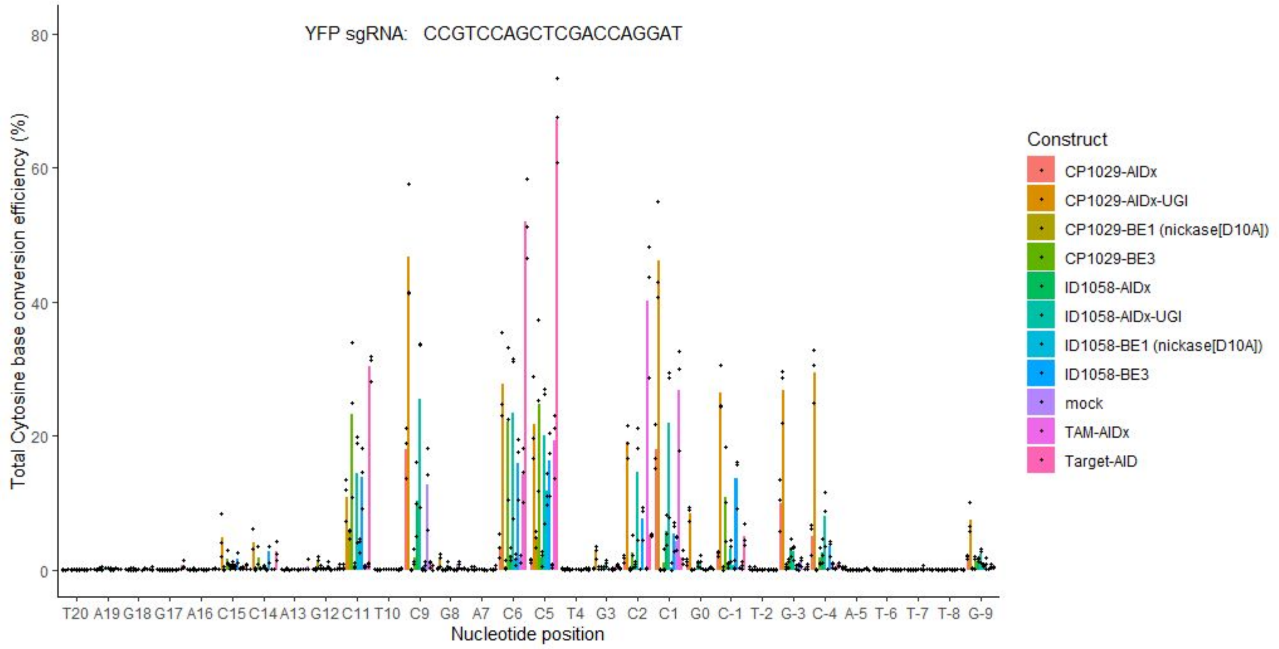

Supplementary Figure 6.

SpCas9 permutants showing both circularly permuted and intradomain ABEmax and miniABEmax variants. Off-target profiling was considered at the highest-edited adenine position in the *MTA2*, *PTBP2*, *LCMT1*, *SCAP, DNAJB* and *SAP30BP* transcripts, as described in **Methods**. MiSeq data derived from nine independent replicates with sgRNAs targeting the *ABE16*, *YFP*, or non-targeting loci (n=3 biological replicates with three respective technical replicates). Boxplots display the interquartile range; upper and lower hinges correspond to the first and third quartile (25 and 75th percentile) centred around the mean.

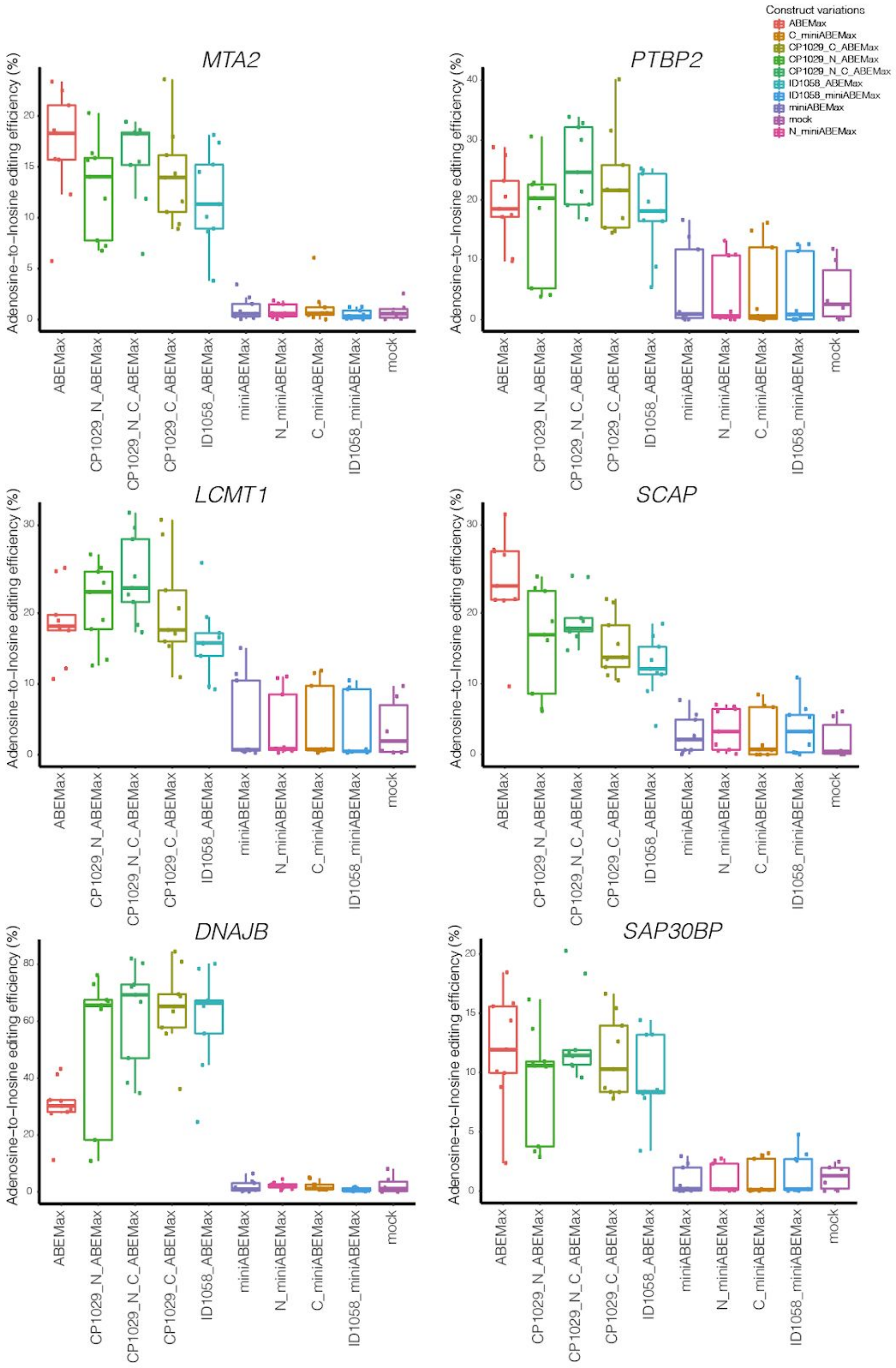

Supplementary Figure 7.

Heatmap showing the localized, off-target profile of SpCas9 ABEmax permutants across promiscuous RNA transcripts. Average editing across the transcript was considered for adenosine-to-inosine (guanine), as described in **Methods**, including that of the position of the highest-edited adenine in the amplicon. Results display the average adenosine to inosine editing at each adenosine nucleotide position within the amplicon and scaled accordingly using the 'superheat' package in R (v. 1.2.5019) . MiSeq data was pooled across nine independent replicates.

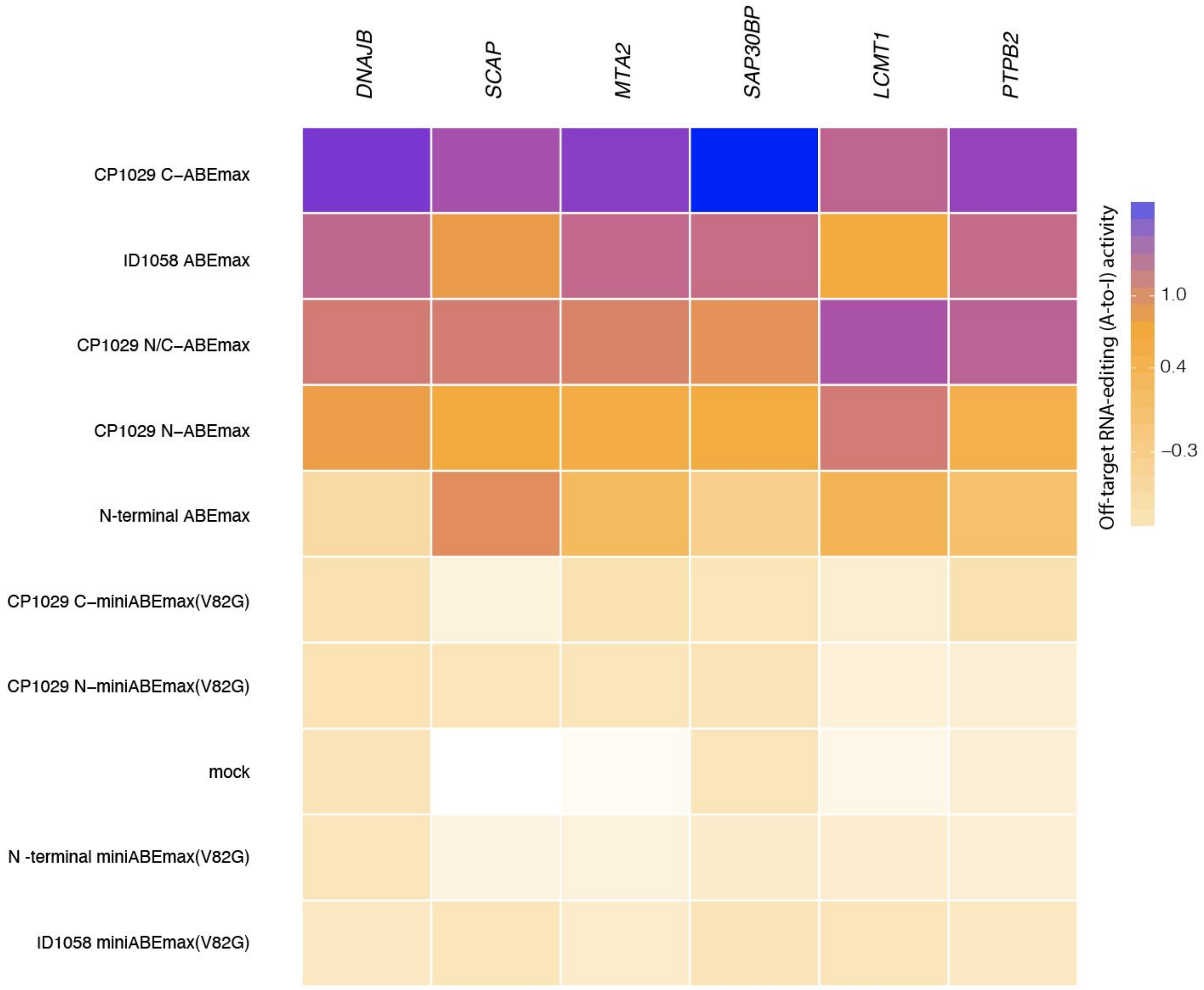

Supplementary Figure 8.

On-target editing efficiencies for various SpCas9 ABEmax permutants with sgRNA targeting the *ABE16* locus. Results are presented as the mean values from MiSeq sequencing across three independent technical replicates.

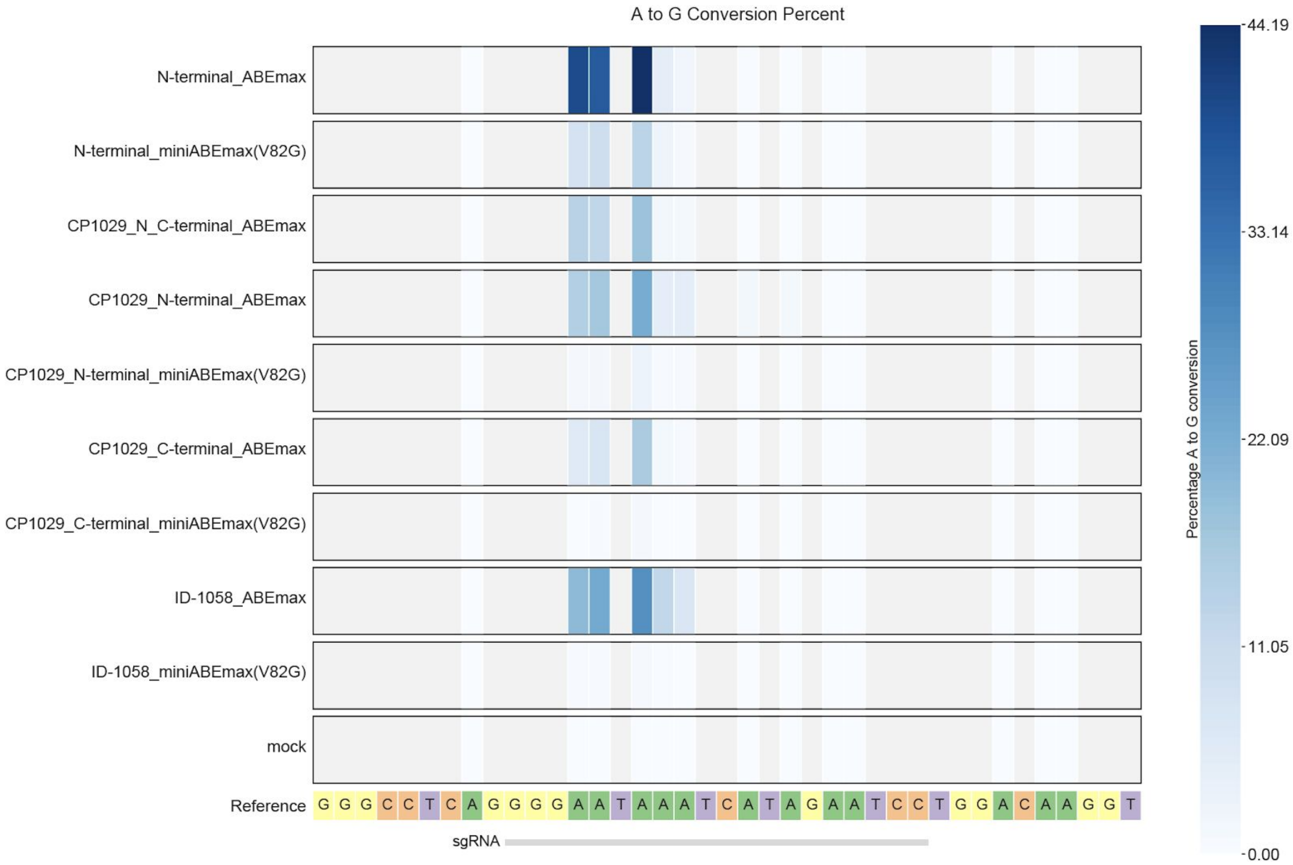

Supplementary Figure 9.

On-target editing efficiencies for various SpCas9 ABEmax permutants with sgRNA targeting the *YFP* locus. Results are presented as the mean values from MiSeq sequencing across three independent technical replicates.

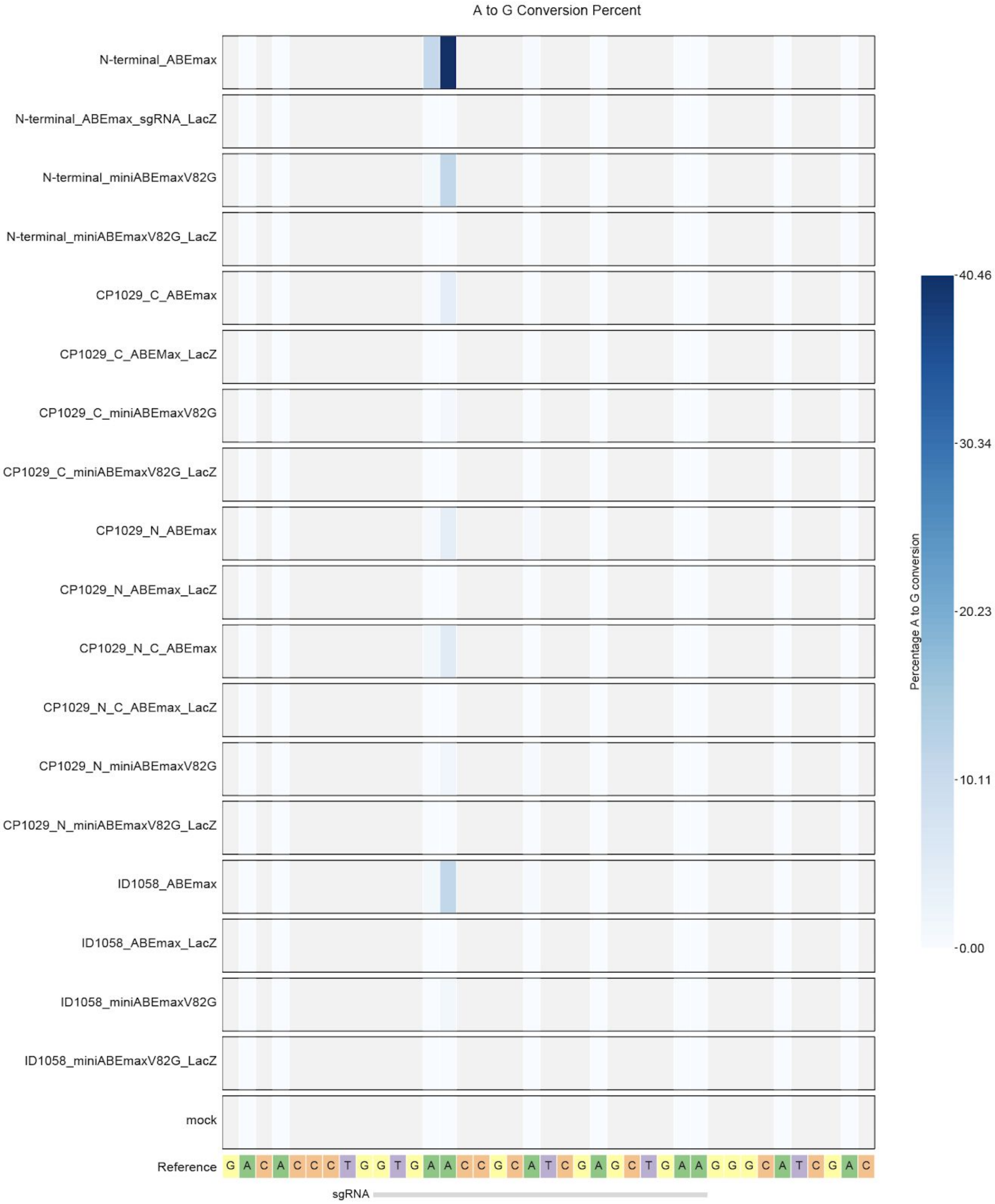

Supplementary Figure 10.

Engineering and profiling of a microABE. Surface structural representations of *S. aureus* Cas9 (purple; PDB:5CZZ) with the corresponding target DNA (red) and gRNA (orange) complex. Residues marked in yellow or green, demarcate amino acid positions 119-132 or 730-745, respectively. All graphical representations generated with PyMol (Methods).

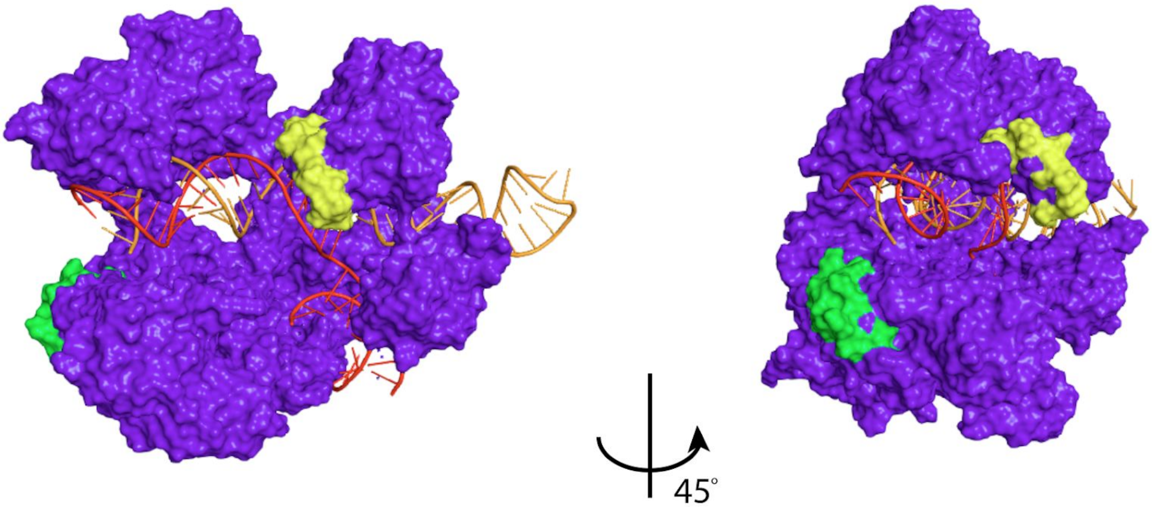

Supplementary Figure 11.

Base editing activity for each intradomain inserted miniABEmax (V82G) Sacas9 tested. The SaCas9n amino acid insertion site is displayed, and each datapoint represents the mean across three technical replicates. A nickase SaCas9 with an N-terminal linked ABEmax and miniABEmax (V82G) was used for direct comparison.

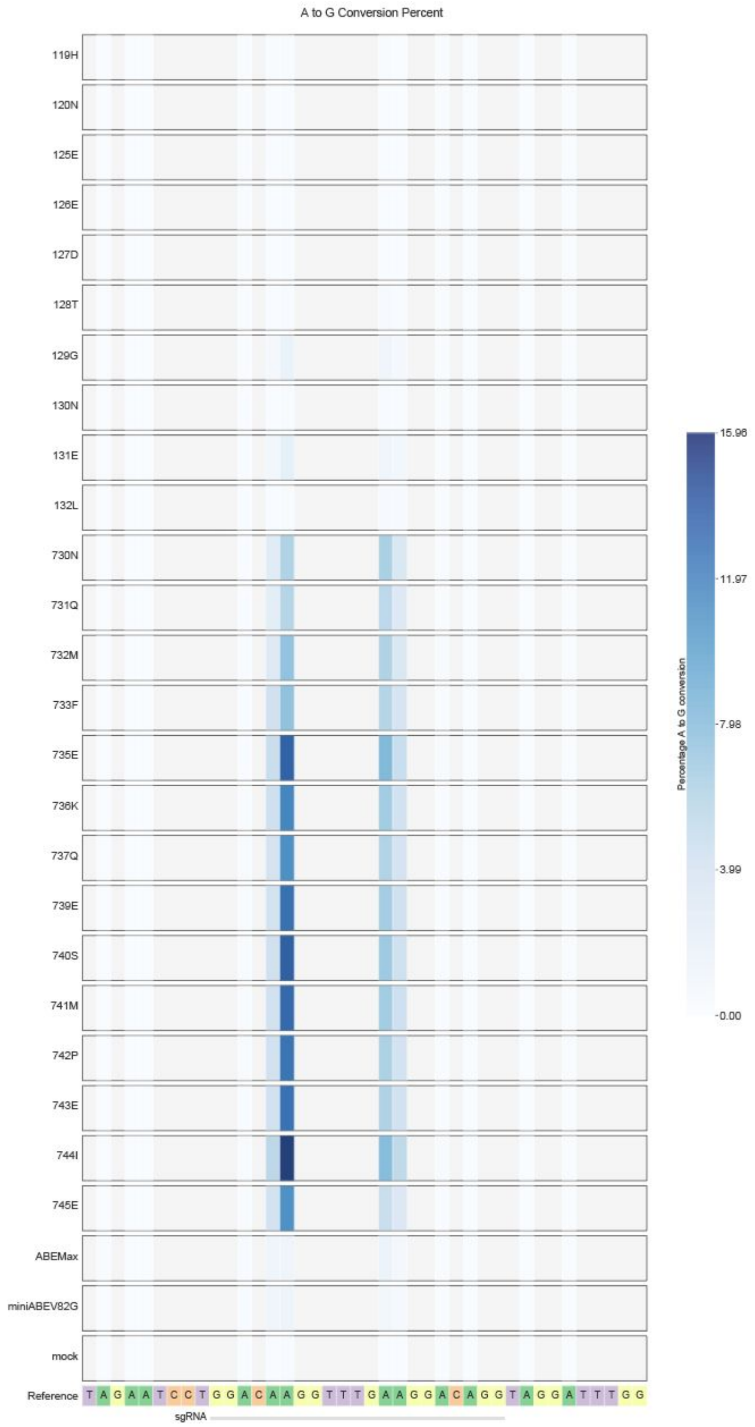

Supplementary Figure 12.

On-target activity window of intradomain inserted miniABEmax (V82G) nSacas9 constructs compared to N-terminal linked ABEmax or miniABEmax (V82G). Editing efficiency was determined at various adenine positions in a 21-nucleotide activity window derived from a composite of MiSeq data generated by sgRNAs targeting the *ABE16* and *RNF2* loci. Individual data points are shown for each construct at each nucleotide position across two independent biological replicate sgRNA sites (*ABE16* and *RNF2*) (n=3 technical replicates for each sgRNA site performed on independent days).

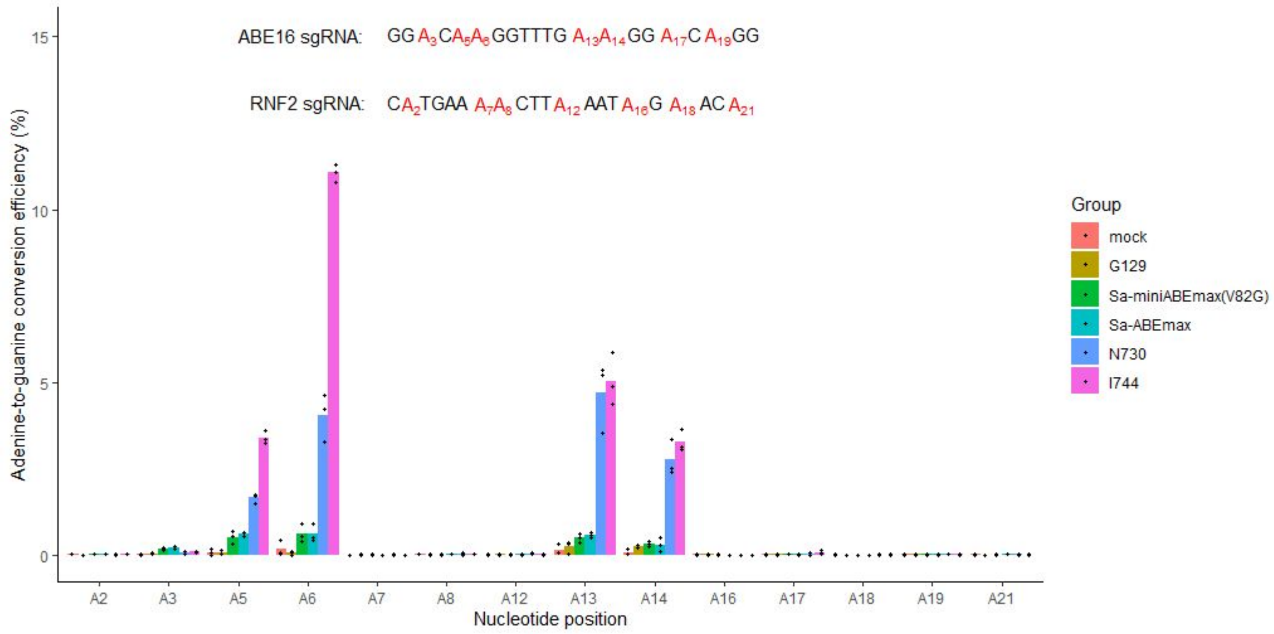

Supplementary Figure 13.

Cytosine deaminase efficiencies for SaCas9n intradomain inserted hAIDx. hAIDx protein domain was inserted at residue G129, N730 and I744 in SaCas9n. sgRNAs targeting the HEKsite4, ABE5, and ABE9 sites were used. Heatmap data points are presented as the average over three technical replicates.

HEKsite4:

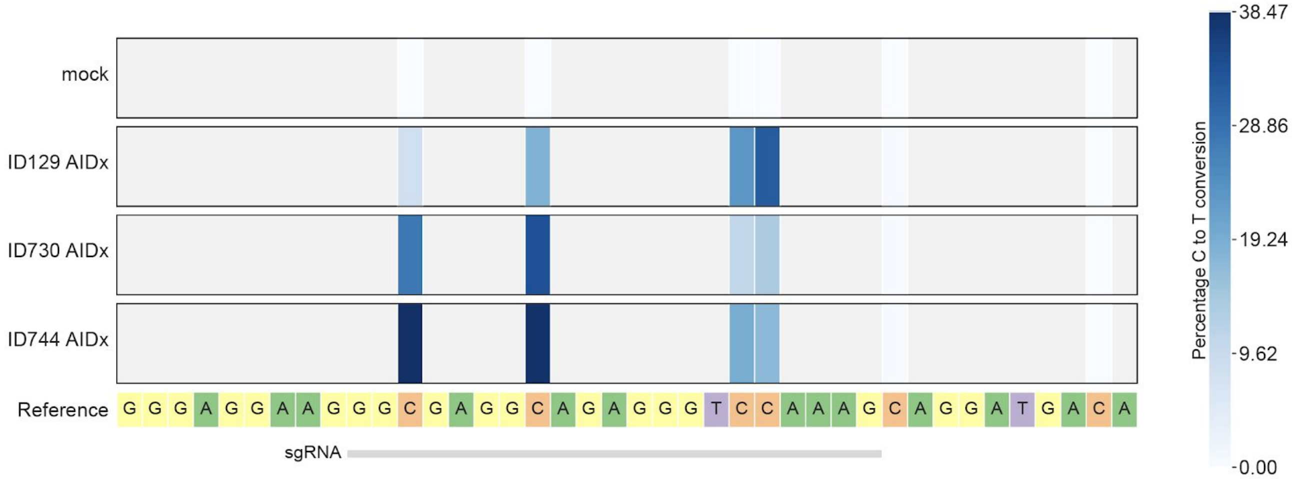

ABE5:

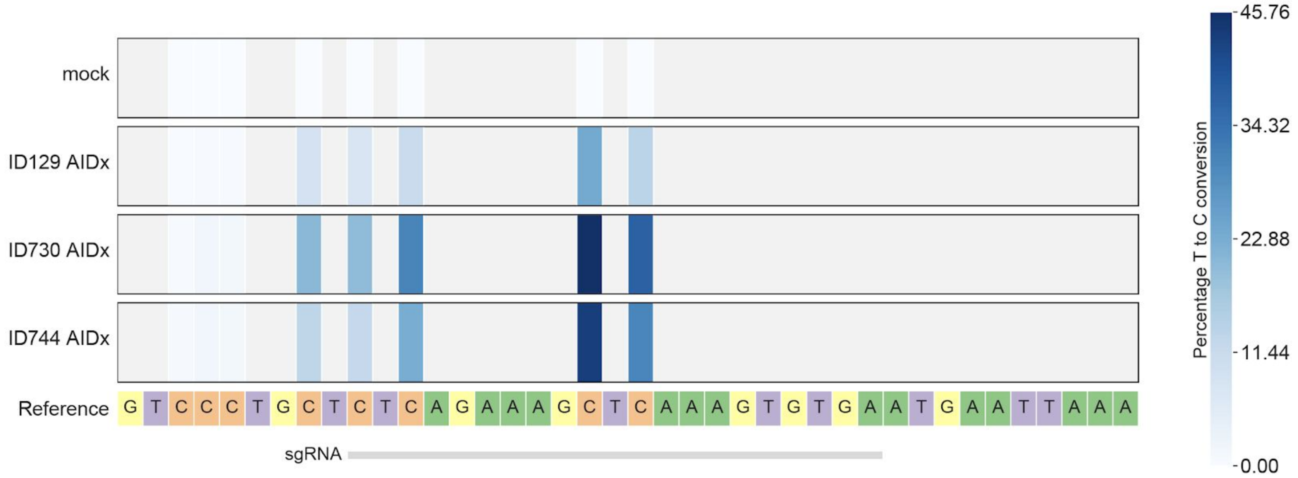

ABE9:

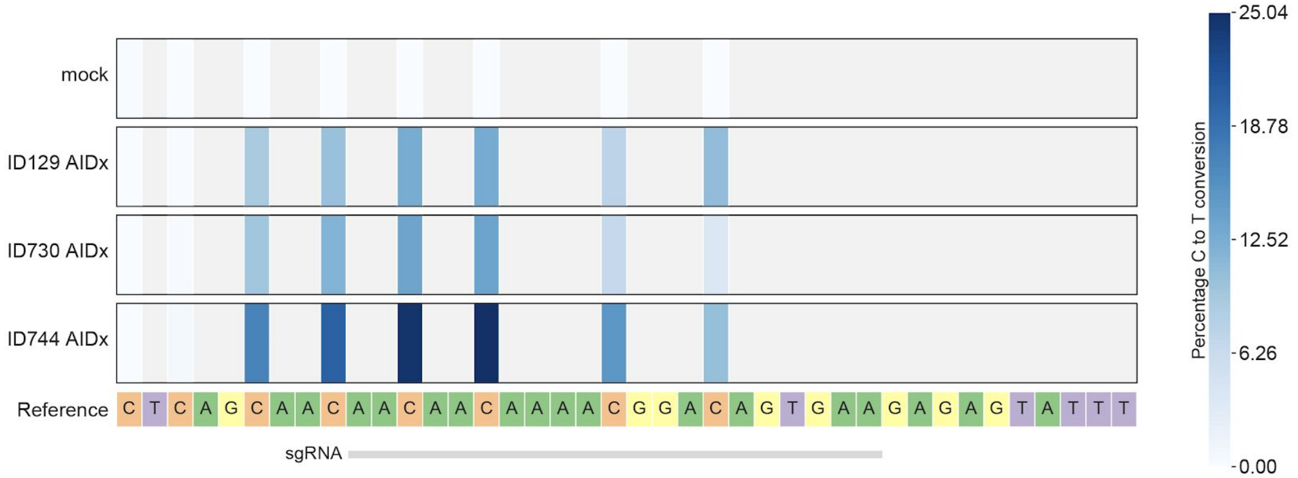

Supplementary Figure 14.

Off-target profiling of Sa-ABEmax, Sa-miniABEmax(V82G), G129, N730, and I744 (microABE I744). Data were generated from nine independent replicates using sgRNA targeting *ABE16, YFP,* and non-targeting *LacZ*. Off-target editing was considered at the position of the highest-edited adenine nucleotide in the *MTA2*, *PTBP2*, *LCMT1*, *SCAP, DNAJB* and *SAP30BP* transcripts, as described in **Methods**. Boxplots display the interquartile range; upper and lower hinges correspond to the first and third quartile (25 and 75th percentile) centred around the mean.

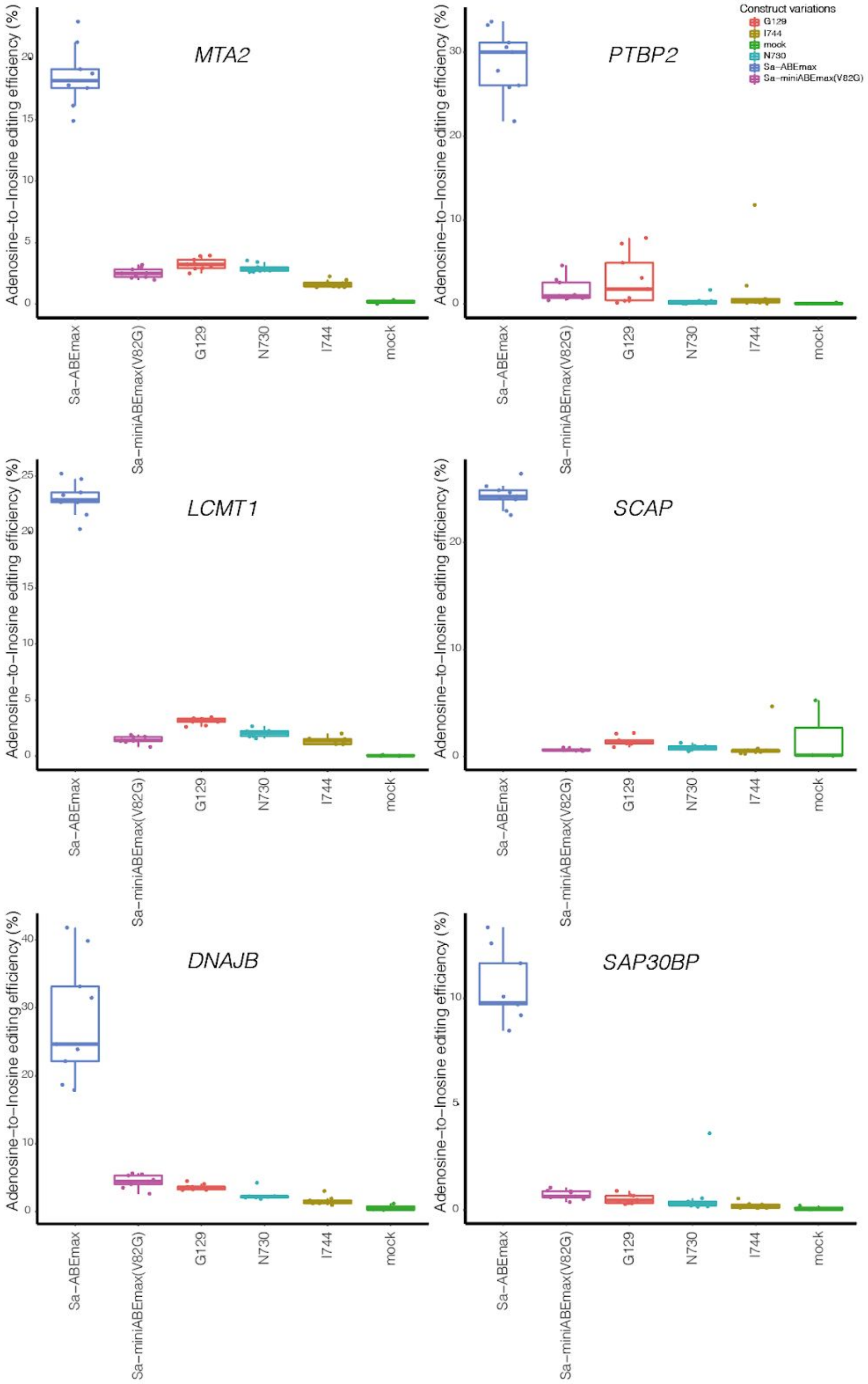

Supplementary Figure 15.

Heatmap showing the localized, off-target profile of SaCas9 ABE permutants across promiscuous RNA transcripts. Average editing across the transcript was considered for adenosine-to-inosine (guanine), as described in **Methods**, including that of the position of the highest-edited adenine in the amplicon. MiSeq data was pooled across nine independent replicates.

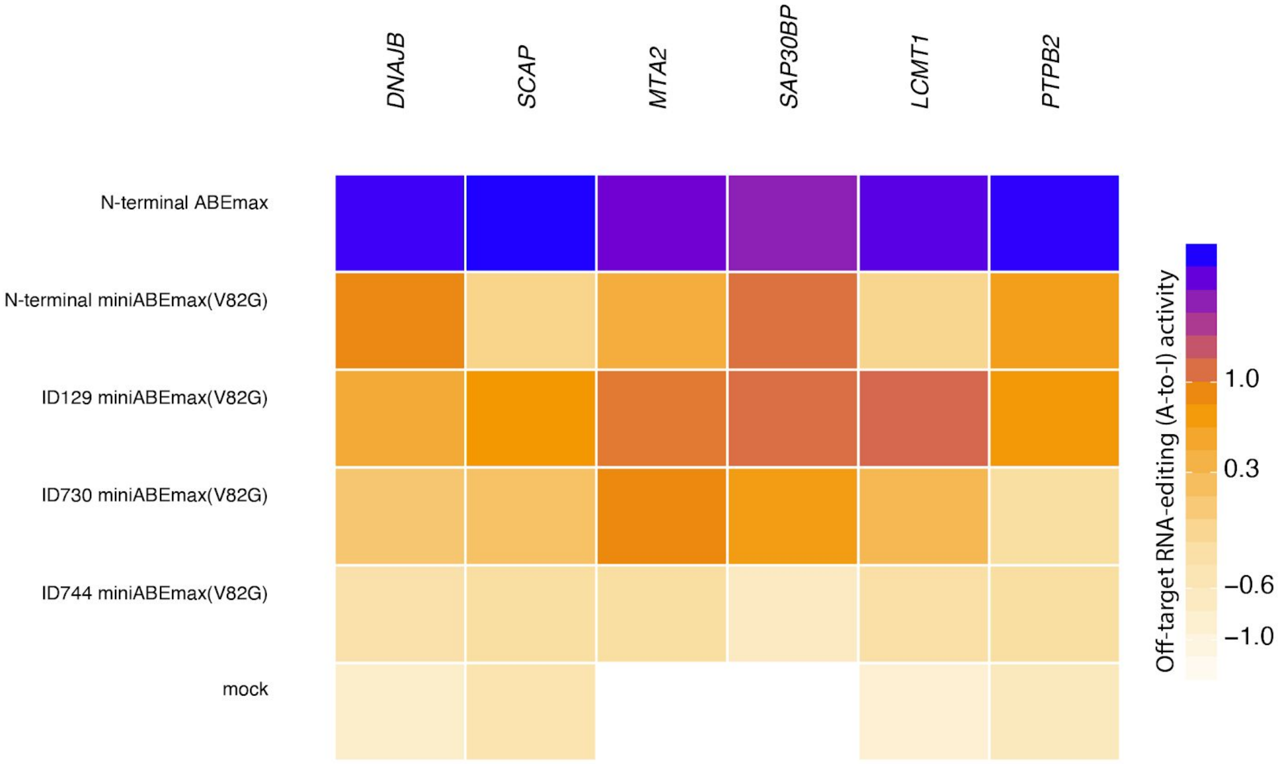

Supplementary Figure 16.

Uncropped images of western blot gels used to demonstrate immuno-binding of antibodies targeting the N- and C-terminal of SaCas9, for each base editor constructed tested. Relative densitometry for both the N- and C-terminal SaCas9 antibodies to histone H3 was calculated by dividing the chemiluminescent intensity for the protein band-of-interest by the signal for the corresponding loading control for each well.

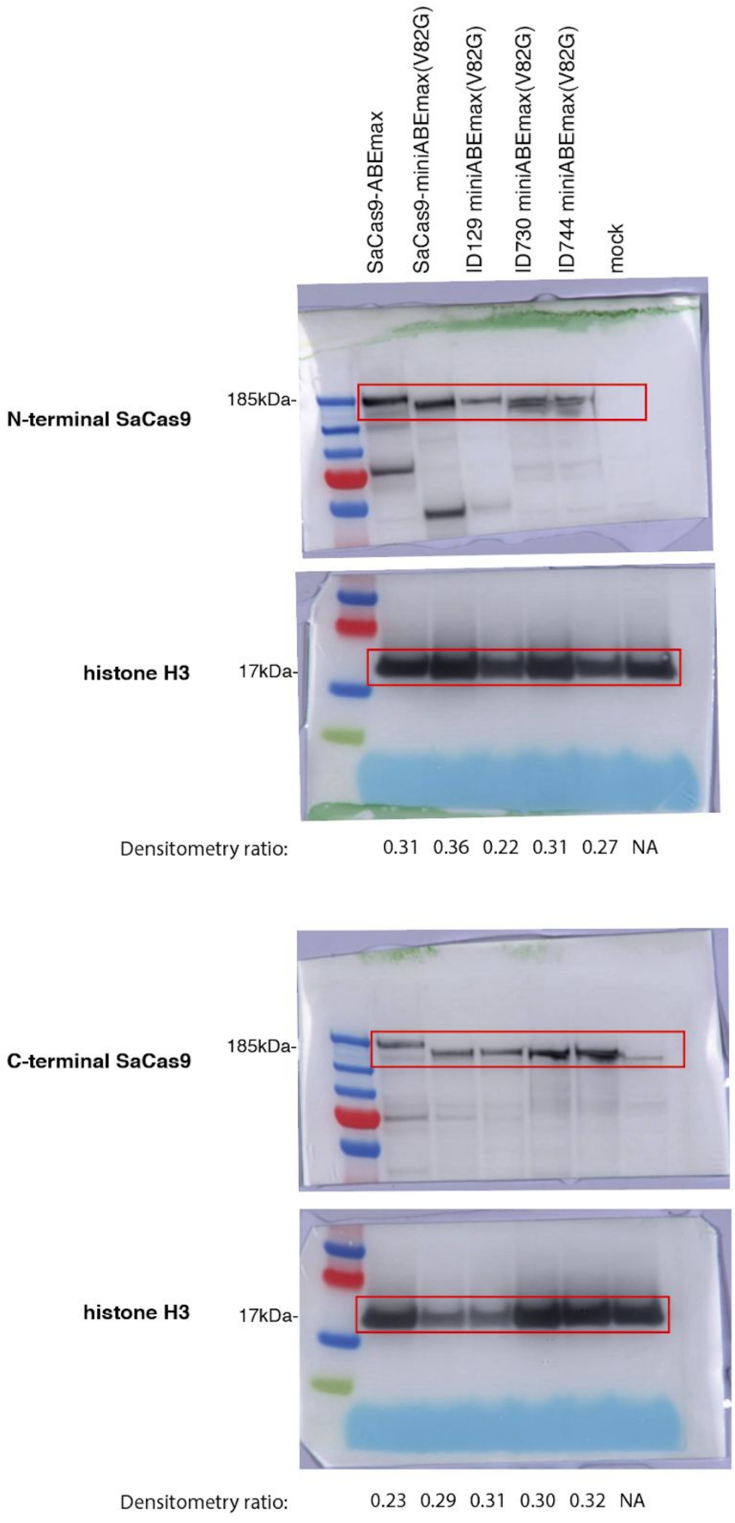

Supplementary Figure 17.

Transcriptomic profiling of microABE I744 (ID744 miniABEmax [V82G]), Sa-ABEmax (N-terminal ABEmax), and Sa-miniABEmax (V82g) (N-terminal miniABEmax [V82G]) showing the number of reads containing adenosine-to-inosine editing in HEK293A-YFP cells. Number of reads showing adenosine-to-inosine edits in the transcriptome is arranged by mapped alignments to their chromosomal position (n=3 independent pooled technical replicates harvested on separate days; refer to **Methods RNA-seq analysis**).

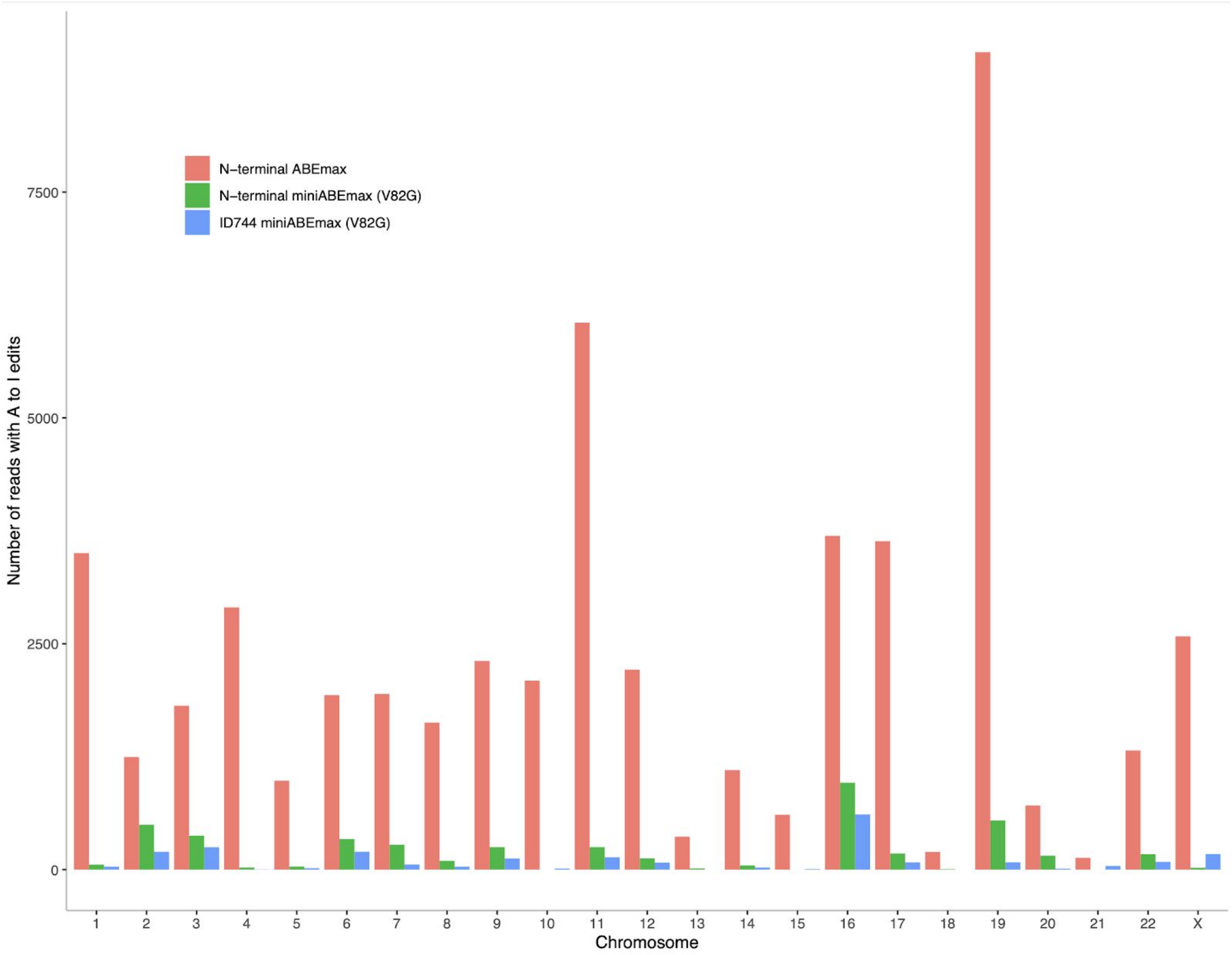

Supplementary Figure 18.

Editing profile for the nSaCas9 N-terminal linked Sa-ABEmax, Sa-miniABEmax(V82G) and their nSaCas9-KKH counterparts compared to the microABE I744 (intradomain-SaCas9n I744) with a sgRNA targeting the *PCDH15* Arg245Ter (NM_033056.4:c.733C>T) variant or *LacZ*. The disease causing variant being targeted is demarcated by the red rectangle. Heatmap data points are presented as the average over three technical replicates. Nucleotide position is shown for the antisense strand whereby a thymine-to-cytosine transition indicates adenine-to-guanine editing.

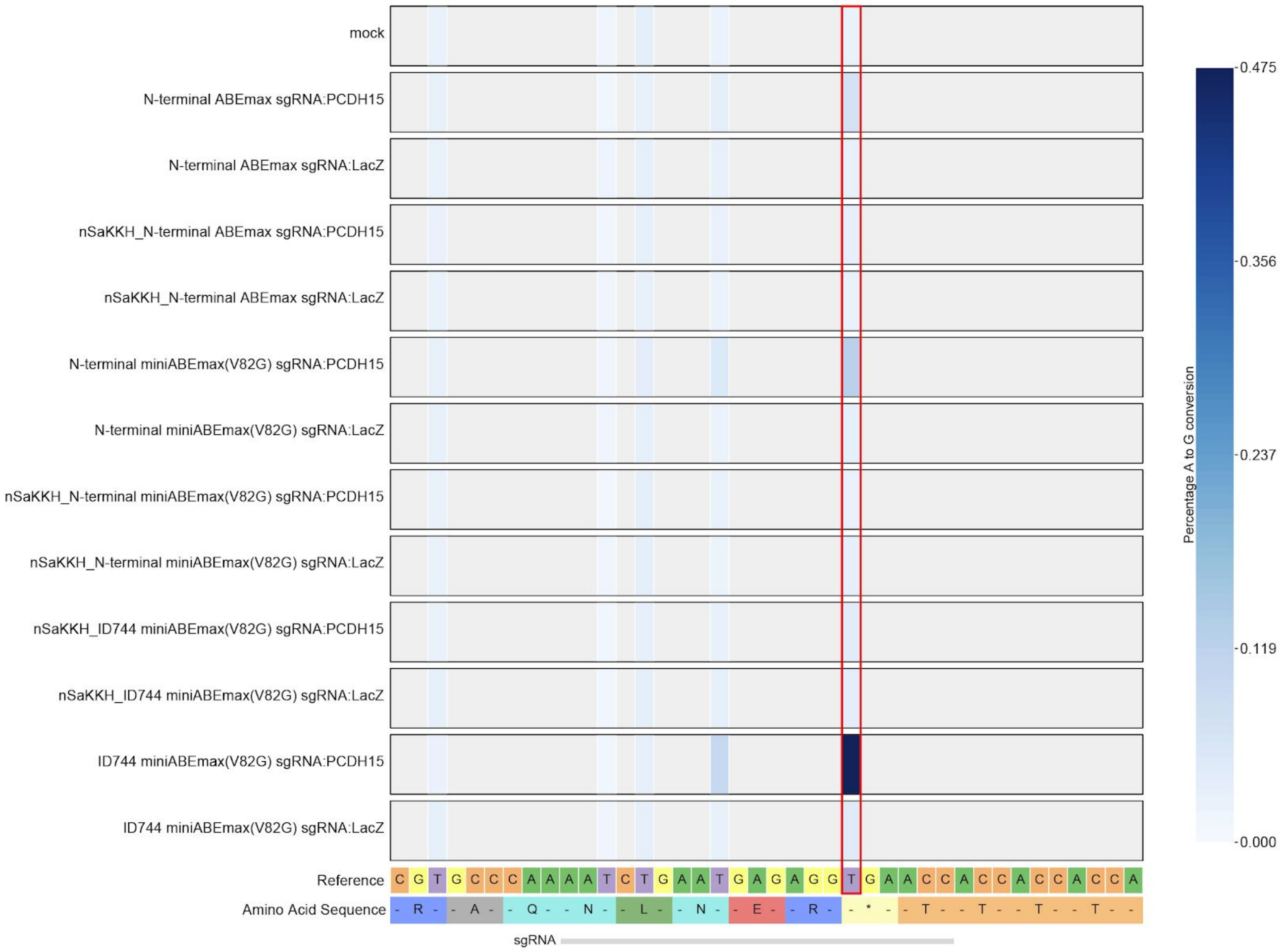

Supplementary Figure 19.

Heatmap showing the incidence of off-target events at several promiscuous RNA transcripts for constructs targeting the *PCDH15* Arg245Ter variant. Data is generated from targeted MiSeq sequencing averaged across six replicates in an engineered Flp-In TRex 293 cell line expressing the cDNA transcript of *PCDH15*.

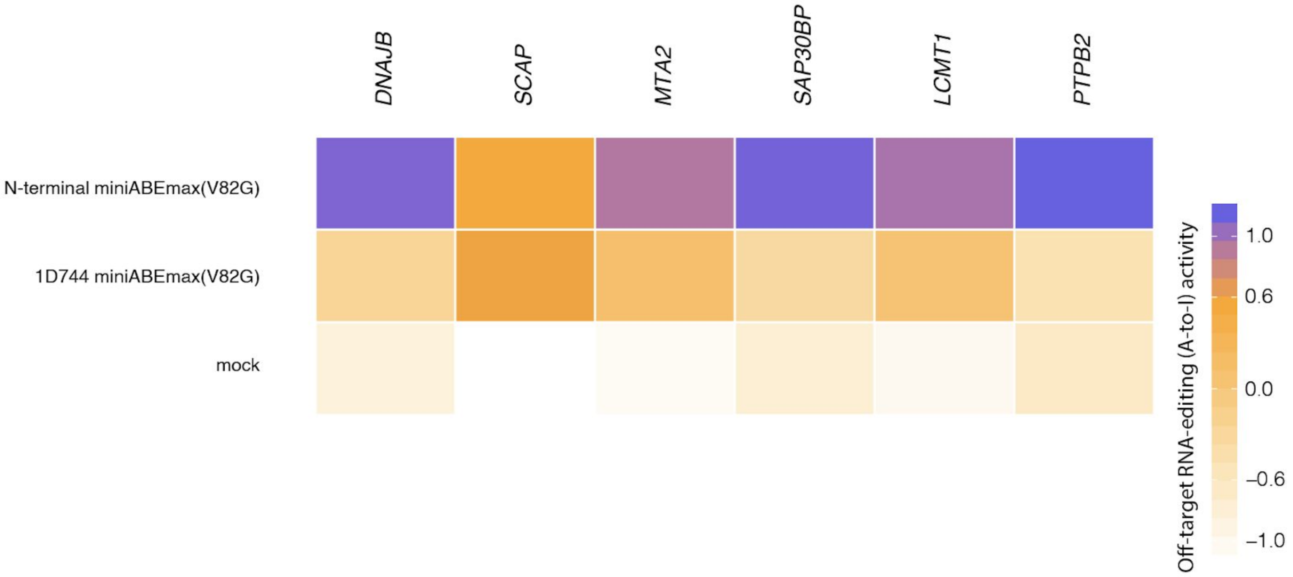

Supplementary Figure 20.

Editing profile in H9-derived retinal organoids for the microABE I744 (intradomain-nSaCas9 I744) with a sgRNA targeting the ABE11 site or *LacZ*, delivered via AAV-7m8 or AAV-DJ. Retinal organoids were transduced with 8.0 x 10^{10} to 1.2 x 10^{11} viral genomes for 7 days without selection. Heatmap data points are presented as the average over two technical replicates.

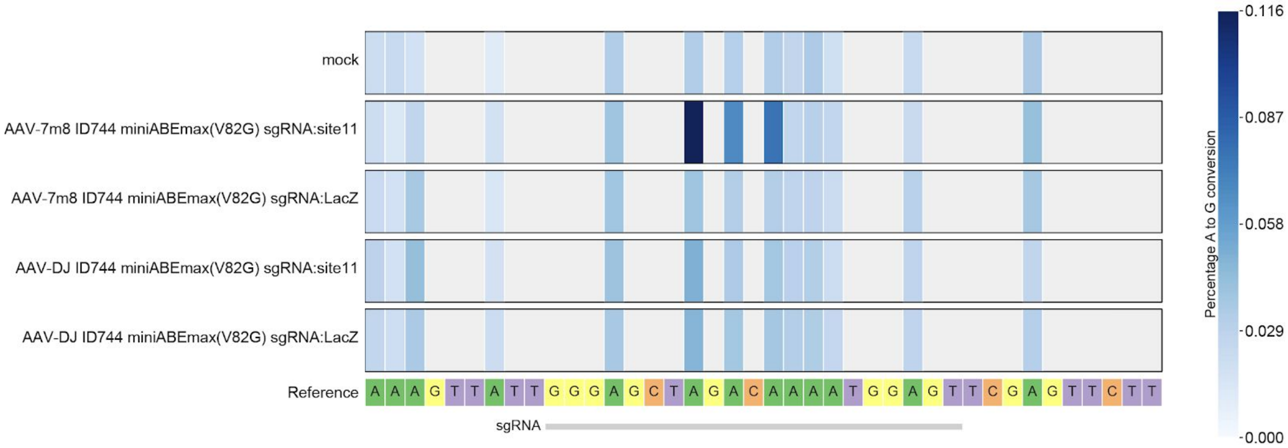

Supplement: Supplementary file 1 — Supplementary Information [file 41467_2020_18715_MOESM1_ESM.pdf]
