# Supplementary material for: Engineering domain-inlaid SaCas9 adenine base editors with reduced RNA off-targets and increased on-target DNA editing
Source: Nat Commun. 2020 Sep 25;11:4871. doi: 10.1038/s41467-020-18715-y (PMC7519688; doi:10.1038/s41467-020-18715-y)
Supplement: Supplementary file 2 — Reporting Summary [file 41467_2020_18715_MOESM2_ESM.pdf]

nature research

Corresponding author(s): Minh Nguyen Tran; Alex Hewitt

Last updated by author(s): Sep 10, 2020

Reporting Summary

Nature Research wishes to improve the reproducibility of the work that we publish. This form provides structure for consistency and transparency in reporting. For further information on Nature Research policies, see Authors & Referees and the Editorial Policy Checklist .

Statistics

For all statistical analyses, confirm that the following items are present in the figure legend, table legend, main text, or Methods section.

n/a	Confirmed	
☐	☒	The exact sample size (*n*) for each experimental group/condition, given as a discrete number and unit of measurement
☐	☒	A statement on whether measurements were taken from distinct samples or whether the same sample was measured repeatedly
☐	☒	The statistical test(s) used AND whether they are one- or two-sided<br>*Only common tests should be described solely by name; describe more complex techniques in the Methods section.*
☒	☐	A description of all covariates tested
☐	☒	A description of any assumptions or corrections, such as tests of normality and adjustment for multiple comparisons
☐	☒	A full description of the statistical parameters including central tendency (e.g. means) or other basic estimates (e.g. regression coefficient) AND variation (e.g. standard deviation) or associated estimates of uncertainty (e.g. confidence intervals)
☐	☒	For null hypothesis testing, the test statistic (e.g. *F*, *t*, *r*) with confidence intervals, effect sizes, degrees of freedom and *P* value noted<br>*Give P values as exact values whenever suitable.*
☒	☐	For Bayesian analysis, information on the choice of priors and Markov chain Monte Carlo settings
☒	☐	For hierarchical and complex designs, identification of the appropriate level for tests and full reporting of outcomes
☒	☐	Estimates of effect sizes (e.g. Cohen's *d*, Pearson's *r*), indicating how they were calculated

Our web collection on statistics for biologists contains articles on many of the points above.

Software and code

Policy information about availability of computer code

Data collection	Next-generation sequencing data was collected with MiSeq (targeted amplicon sequencing) using a V3 600 Paired-end flow cell and RNA-seq performed using a HiSeq X Ten to capture 2X150-bp PE reads.
Data analysis	PyMol v2.3.1, Crispresso2, trimmomatic-0.39, fastq-join v1.3.1-2, R v3.6.1

For manuscripts utilizing custom algorithms or software that are central to the research but not yet described in published literature, software must be made available to editors/reviewers. We strongly encourage code deposition in a community repository (e.g. GitHub). See the Nature Research guidelines for submitting code & software for further information.

Data

Policy information about availability of data

All manuscripts must include a data availability statement. This statement should provide the following information, where applicable:

- Accession codes, unique identifiers, or web links for publicly available datasets
- A list of figures that have associated raw data
- A description of any restrictions on data availability

All editor plasmids have been submitted to Addgene for public release. All raw sequencing reads have been uploaded to the European Nucleotide Archive under the accessions: PRJEB35675 (MiSeq sequencing: https://www.ebi.ac.uk/ena/browser/view/PRJEB35675); PRJEB38819 (RNAseq profiling: https://www.ebi.ac.uk/ena/browser/view/PRJEB38819); and PRJEB38622 (whole exome sequencing: https://www.ebi.ac.uk/ena/browser/view/PRJEB38622).
Crystal structures for SpCas9 and SaCas9 are available from the Protein Data Bank at https://www.rcsb.org/structure/4oo8 and https://www.rcsb.org/structure/5CZZ, respectively. Source data are provided with this paper

Field-specific reporting

Please select the one below that is the best fit for your research. If you are not sure, read the appropriate sections before making your selection.

- [x] Life sciences
- [ ] Behavioural & social sciences
- [ ] Ecological, evolutionary & environmental sciences

For a reference copy of the document with all sections, see nature.com/documents/nr-reporting-summary-flat.pdf

Life sciences study design

All studies must disclose on these points even when the disclosure is negative.

Sample size	For off-target analyses, three biological triplicates were used, resulting in an n=9. Three independent biological triplicates were chosen based upon a previous publication analysing RNA off-target events (https://doi.org/10.1038/s41587-019-0236-6). For on-target determination of the activity window, at the time, more than 15 sgRNA target sites were selected based on previous literature at the time (https://doi.org/10.1038/s41586-018-0070-x). All samples used technical triplicates performed on different days using different cell passages.
Data exclusions	No data was excluded
Replication	Biological and technical replicates were performed using fresh aliquots of cells from different passages over the course of days to several months. For each biological replicate, triplicate technical replicates were performed on separate days. Biological replicates were performed in parallel. All attempts were successful, and no experiment needed to be repeated. A different cell-line was used to validate key findings and those experiments were performed by up to three different researchers.
Randomization	Samples were not randomized. Covariates were controlled for by running controls in parallel during each experiment. Downstream processing of each sample was also performed simultaneously wherein sample sets were kept together. For example, during the transfection of a construct for one particular technical replicate, replicates were harvested, processed, and sequenced on the same MiSeq V3 flow cell. At no point were any samples belonging to a particular replicate processed separately.
Blinding	Blinding was not performed based on previous literature in the field at the time (https://doi.org/10.1038/s41587-019-0236-6)

Reporting for specific materials, systems and methods

We require information from authors about some types of materials, experimental systems and methods used in many studies. Here, indicate whether each material, system or method listed is relevant to your study. If you are not sure if a list item applies to your research, read the appropriate section before selecting a response.

Materials & experimental systems

n/a	Involved in the study	
[ ]	[x]	Antibodies
[ ]	[x]	Eukaryotic cell lines
[x]	[ ]	Palaeontology
[x]	[ ]	Animals and other organisms
[x]	[ ]	Human research participants
[x]	[ ]	Clinical data

Methods

n/a	Involved in the study	
[x]	[ ]	ChIP-seq
[x]	[ ]	Flow cytometry
[x]	[ ]	MRI-based neuroimaging

Antibodies

Antibodies used	H3pan monoclonal antibody (clone 1B1B2), S. aureus CRISPR/Cas9 monoclonal antibody (C15200230), DYKDDDDK Tag Antibody (MA1-91878-D550), Mouse IgG (H+L) Secondary Antibody (31430)

Validation	H3pan monoclonal antibody (clone 1B1B2) has been validated by Diagenode via Western blot and ChIP analysis (https://www.diagenode.com/files/products/antibodies/Datasheet_H3pan_C15200011.pdf)<br><br>S. aureus CRISPR/Cas9 monoclonal antibody validated in manuscript, also please see: https://www.diagenode.com/files/products/antibodies/C15200230-aureus-cas9-monoclonal.pdf<br><br>DYKDDDDK Tag Antibody (MA1-91878-D550) validated by manufacturer (https://www.thermofisher.com/antibody/product/DYKDDDDK-Tag-Antibody-clone-FG4R-Monoclonal/MA1-91878-D550#!%2F%23references-heading)<br><br>Mouse IgG (H+L) Secondary Antibody (31430) validated by manufacturer (https://www.thermofisher.com/antibody/product/Goat-anti-Mouse-IgG-H-L-Secondary-Antibody-Polyclonal/31430)

Eukaryotic cell lines

Policy information about cell lines

Cell line source(s)	HEK293A-YFP cells were generated as previously described (doi: 10.1167/iovs.16-19316), which were in turn derived from HEK293A (R70507, ThermoFisher Scientific), Flp-In TRex 293 (R78007, Invitrogen, ThermoFisher Scientific), and H9 pluripotent stem cells (https://www.wicell.org/home/stem-cells/catalog-of-stem-cell-lines/wa09.cmsx)
Authentication	HEK293A-YFP cells were authenticated in a previously described study (doi: 10.1167/iovs.16-19316). HEK293A cells underwent STR profiling (AMEL, CSF1PO, D5S818, D7S820, D13S317, D16S539, D21S11, TH01, TPOX, and vWA) through the Australian Genome Research Facility LTD, VIC, Australia; Flp-In TRex 293 and H9 cell lines were authenticated by supplier.
Mycoplasma contamination	Flp-In TRex 293 and HEK293A-YFP cells were tested. H9 pluripotent stem cells were not tested. Supernatant was analyzed every two weeks using MPCR Mycoplasma Test Kit I/C (Banksia Scientific). Both cell lines continuously tested negative.
Commonly misidentified lines<br>(See ICLAC register)	HEK293A, Flp-In TRex 293, and H9 cell lines are not listed in the ICLAC register (Version 9)
