# Supplementary material for: Engineering domain-inlaid SaCas9 adenine base editors with reduced RNA off-targets and increased on-target DNA editing
Source: Nat Commun. 2020 Sep 25;11:4871. doi: 10.1038/s41467-020-18715-y (PMC7519688; doi:10.1038/s41467-020-18715-y)
Supplement: Supplementary file 4 — Supplementary Data 2 [file 41467_2020_18715_MOESM4_ESM.pdf]

Amino acid sequence of microABE I744, SaCas9-miniABEmax (V82G), SaCas9-ABEmax, microAIDx I744

Pink=Kozak sequence
Grey=SaCas9 (D10A)
Blue=nuclear localization sequence
Orange=44-amino-acid linker
Green=evolved ecTadA monomer
Red=V82G amino acid substitution
Yellow=wildtype ecTadA monomer
Dark green=hAIDx
Light magenta=UGI
Cyan=20-amino-acid glycine-serine linker
Cornflower blue=32 amino-acid linker
Berry=P2A
Purple=Blasticidin-S-deaminase

SaCas9-ABEMax
-gccacc-MKRTADGSEFESPKKKRKVSEVEFSHEYWMRHALTLAKRAWDEREVPVGAVLVHNNRVIGEGWNRPIGRHDPT
AHAEIMALRQGGLVMQNYRLIDATLYVTLEPCVMCAGAMIHSRIGRVVFGARDAKTGAAGSLMDVLHHPGMNHRVEIT
EGILADECAALLSDFFRMRRQEIKAQKKAQSSTDSGGSSGGSSGSETPGTSESATPESSGGSSGGSSEVEFSHEYWMRHALT
LAKRARDEREVPVGAVLVLNNRVIGEGWNRAIGLHDPTAHAEIMALRQGGLVMQNYRLIDATLYVTFEPCVMCAGAMIH
SRIGRVVFGVRNAKTGAAGSLMDVLHYPGMNHRVEITEGILADECAALLCYFFRMPRQVFNAQKKAQSSTDSGGSSGGSS
GSETPGTSESATPESSGGSSGGSGKRNYILGLAIGITSVGYGIIDYETRDVIDAGVRLFKEANVENNEGRRSKRGARRLKRRRR
HRIQRVKKLLFDYNLLTDHSELSGINPYEARVKGLSQKLSEEEFSAALLHLAKRRGVHNVNEVEEDTGNELSTKEQISRNSKAL
EEKYVAELQLERLKKDGEVRGSINRFKTSDYVKEAKQLLKVQKAYHQLDQSFIDTYIDLLETRRTYYEGPGEGSPFGWKDIKE
WYEMLMGHCTYFPEELRSVKYAYNADLYNALNDLNNLVITRDENEKLEYYEKFQIIENVFKQKKKPTLKQIAKEILVNEEDIK
GYRVTSTGKPEFTNLKVYHDIKDITARKEIIENAELLDQIAKILTIYQSSEDIQEELTNLNSELTQEEIEQISNLKGYTGTHNLSLKA
INLILDELWHTNDNQIAIFNRLKLVPKKVDLSQQKEIPTTLVDDFILSPVVKRSFIQSIKVINAIIKKYGLPNDIIIELAREKNSKDA
QKMINEMQKRNRQTNERIEEIIRTTGKENAKYLIEKIKLHDMQEGKCLYSLEAIPLEDLLNNPFNYEVDHIIPRSVSFDNSFNN
KVLVKQEENSKKGNRTPFQYLSSSDSKISYETFKKHILNLAKGKGRISKTKKEYLLEERDINRFSVQKDFINRNLVDTRYATRGL
MNLLRSYFRVNNLDVKVKSINGGFTSFLRRKWKFKKERNKGYKHHAEDALIIANADFIFKEWKKLDKAKKVMENQMFEEK
QAESMPEIETEQEYKEIFITPHQIKHIKDFKDYKYSHRVDKKPNRELINDTLYSTRKDDKGNTLIVNNLNGLYDKDNDKLKKLI
NKSPEKLLMYHHDPQTYQKLKLIMEQYGDEKNPLYKYYEETGNYLTKYSKKDNGPVIKKIKYYGNKLNAHLDITDDYPNSRN
KVVKLSLKPYRFDVYLDNGVYKFVTVKNLDVIKKENYYEVNSKCYEEAKKLKKISNQAEFIASFYNNDLIKINGELYRVIGVNN
DLLNRIEVNMIDITYREYLENMNDKRPPRIIKTIASKTQSIKKYSTDILGNLYEVKSKKHPQIIKKGSGGSPKKKRKVSGGSKRPA
ATKKAGQAKKKKDYKDDDDKGSGATNFSLLKQAGDVEENPGPMAKPLSQEESTLIERATATINSIPISEDYSVASAALSSDG
RIFTGVNVYHFTGGPCAELVVLGTAAAAAGNLTCIVAIGNENRGILSPCGRCRQVLLDLHPGIKAIVKDSDGQPTAVGIREL
LPSGYVWEG*

miniABEmax (V82G)-SaCas9

-gccacc-MKRTADGSEFESPKKKRKVSEVEFSHEYWMRHALTLAKRARDEREVPVGAVLVLNNRVIGEGWNRAIGLHDPTA
HAEIMALRQGGLVMQNYRLIDATLYGTFEPCVMCAGAMIHSRIGRVVFGVRNAKTGAAGSLMDVLHYPGMNHRVEITE
GILADECAALLCYFFRMPRQVFNAQKKAQSSTDSGGSSGGSSGSETPGTSESATPESSGGSSGGSGKRNYILGLAIGITSVGY
GIIDYETRDVIDAGVRLFKEANVENNEGRRSKRGARRLKRRRRHRIQRVKKLLFDYNLLTDHSELSGINPYEARVKGLSQKLSE
EEFSAALLHLAKRRGVHNVNEVEEDTGNELSTKEQISRNSKALEEKYVAELQLERLKKDGEVRGSINRFKTSDYVKEAKQLLK
VQKAYHQLDQSFIDTYIDLLETRRTYYEGPGEGSPFGWKDIKEWYEMLMGHCTYFPEELRSVKYAYNADLYNALNDLNNLV
ITRDENEKLEYYEKFQIIENVFKQKKKPTLKQIAKEILVNEEDIKGYRVTSTGKPEFTNLKVYHDIKDITARKEIIENAELLDQIAKI
LTIYQSSEDIQEELTNLNSELTQEEIEQISNLKGYTGTHNLSLKAINLILDELWHTNDNQIAIFNRLKLVPKKVDLSQQKEIPTTL
VDDFILSPVVKRSFIQSIKVINAIIKKYGLPNDIIIELAREKNSKDAQKMINEMQKRNRQTNERIEEIIRTTGKENAKYLIEKIKLH
DMQEGKCLYSLEAIPLEDLLNNPFNYEVDHIIPRSVSFDNSFNNKVLVKQEENSKKGNRTPFQYLSSSDSKISYETFKKHILNLA
KGKGRISKTKKEYLLEERDINRFSVQKDFINRNLVDTRYATRGLMNLLRSYFRVNNLDVKVKSINGGFTSFLRRKWKFKKERN
KGYKHHAEDALIIANADFIFKEWKKLDKAKKVMENQMFEEKQAESMPEIETEQEYKEIFITPHQIKHIKDFKDYKYSHRVDKK
PNRELINDTLYSTRKDDKGNTLIVNNLNGLYDKDNDKLKKLINKSPEKLLMYHHDPQTYQKLKLIMEQYGDEKNPLYKYYEET
GNYLTKYSKKDNGPVIKKIKYYGNKLNAHLDITDDYPNSRNKVVKLSLKPYRFDVYLDNGVYKFVTVKNLDVIKKENYYEVNS
KCYEEAKKLKKISNQAEFIASFYNNDLIKINGELYRVIGVNNDLLNRIEVNMIDITYREYLENMNDKRPPRIIKTIASKTQSIKKYS
TDILGNLYEVKSKKHPQIIKKGSGGSPKKKRKVSGGSKRPAATKKAGQAKKKKDYKDDDDKGSGATNFSLLKQAGDVEENP
GPMAKPLSQEESTLIERATATINSIPISEDYSVASAALSSDGRIFTGVNVYHFTGGPCAELVVLGTAAAAAAGNLTCIVAIGNE
NRGILSPCGRCRQVLLDLHPGIKAIVKDSDGQPTAVGIRELLPSGYVWEG*

MicroABE I744

-gccacc-MKRNYILGLAIGITSVGYGIIDYETRDVIDAGVRLFKEANVENNEGRRSKRGARRLKRRRRHRIQRVKKLLFDYNLLT
DHSELSGINPYEARVKGLSQKLSEEEFSAALLHLAKRRGVHNVNEVEEDTGNELSTKEQISRNSKALEEKYVAELQLERLKKD
GEVRGSINRFKTSDYVKEAKQLLKVQKAYHQLDQSFIDTYIDLLETRRTYYEGPGEGSPFGWKDIKEWYEMLMGHCTYFPEE
LRSVKYAYNADLYNALNDLNNLVITRDENEKLEYYEKFQIIENVFKQKKKPTLKQIAKEILVNEEDIKGYRVTSTGKPEFTNLKV
YHDIKDITARKEIIENAELLDQIAKILTIYQSSEDIQEELTNLNSELTQEEIEQISNLKGYTGTHNLSLKAINLILDELWHTNDNQIA
IFNRLKLVPKKVDLSQQKEIPTTLVDDFILSPVVKRSFIQSIKVINAIIKKYGLPNDIIIELAREKNSKDAQKMINEMQKRNRQTN
ERIEEIIRTTGKENAKYLIEKIKLHDMQEGKCLYSLEAIPLEDLLNNPFNYEVDHIIPRSVSFDNSFNNKVLVKQEENSKKGNRTP
FQYLSSSDSKISYETFKKHILNLAKGKGRISKTKKEYLLEERDINRFSVQKDFINRNLVDTRYATRGLMNLLRSYFRVNNLDVKV
KSINGGFTSFLRRKWKFKKERNKGYKHHAEDALIIANADFIFKEWKKLDKAKKVMENQMFEEKQAESMPEIPKKKRKVGSS
GSPKKKRKVGSDALDDFDLDMLGSDALDDFGGGSSEVEFSHEYWMRHALTLAKRARDEREVPVGAVLVLNNRVIGEGW
NRAIGLHDPTAHAEIMALRQGGLVMQNYRLIDATLYGTFEPCVMCAGAMIHSRIGRVVFGVRNAKTGAAGSLMDVLHYP
GMNHRVEITEGILADECAALLCYFFRMPRQVFNAQKKAQSSTDGGSGGSGGSGGSGGSGGSGGIETEQEYKEIFITPHQIK
HIKDFKDYKYSHRVDKKPNRELINDTLYSTRKDDKGNTLIVNNLNGLYDKDNDKLKKLINKSPEKLLMYHHDPQTYQKLKLIM
EQYGDEKNPLYKYYEETGNYLTKYSKKDNGPVIKKIKYYGNKLNAHLDITDDYPNSRNKVVKLSLKPYRFDVYLDNGVYKFVT
VKNLDVIKKENYYEVNSKCYEEAKKLKKISNQAEFIASFYNNDLIKINGELYRVIGVNNDLLNRIEVNMIDITYREYLENMNDK
RPPRIIKTIASKTQSIKKYSTDILGNLYEVKSKKHPQIIKKGSGGSKRPAATKKAGQAKKKKDYKDDDDKGSGATNFSLLKQAG
DVEENPGPMAKPLSQEESTLIERATATINSIPISEDYSVASAALSSDGRIFTGVNVYHFTGGPCAELVVLGTAAAAAAGNLTCI
VAIGNENRGILSPCGRCRQVLLDLHPGIKAIVKDSDGQPTAVGIRELLPSGYVWEG*

MicroAIDx I744

-gccacc-MKRNYILGLAIGITSVGYGIIDYETRDVIDAGVRLFKEANVENNEGRRSKRGARRLKRRRRHRIQRVKKLLFDYNLLT
DHSELSGINPYEARVKGLSQKLSEEEFSAALLHLAKRRGVHNVNEVEEDTGNELSTKEQISRNSKALEEKYVAELQLERLKKD
GEVRGSINRFKTSDYVKEAKQLLKVQKAYHQLDQSFIDTYIDLLETRRTYYEGPGEGSPFGWKDIKEWYEMLMGHCTYFPEE
LRSVKYAYNADLYNALNDLNNLVITRDENEKLEYYEKFQIIENVFKQKKKPTLKQIAKEILVNEEDIKGYRVTSTGKPEFTNLKV
YHDIKDITARKEIIENAELLDQIAKILTIYQSSEDIQEELTNLNSELTQEEIEQISNLKGYTGTHNLSLKAINLILDELWHTNDNQIA
IFNRLKLVPKKVDLSQQKEIPTTLVDDFILSPVVKRSFIQSIKVINAIIKKYGLPNDIIIELAREKNSKDAQKMINEMQKRNRQTN
ERIEEIIRTTGKENAKYLIEKIKLHDMQEGKCLYSLEAIPLEDLLNNPFNYEVDHIIPRSVSFDNSFNNKVLVKQEENSKKGNRTP
FQYLSSSDSKISYETFKKHILNLAKGKGRISKTKKEYLLEERDINRFSVQKDFINRNLVDTRYATRGLMNLLRSYFRVNNLDVKV
KSINGGFTSFLRRKWKFKKERNKGYKHHAEDALIIANADFIFKEWKKLDKAKKVMENQMFEEKQAESMPEIPKKKRKVGSS
GSPKKKRKVGSDALDDFDLDMLGSDALDDFGGGSMDSLLMNRRKFLYQFKNVRWAKGRRETYLCYVVKRRDSATSFSLD
FGYLRNKNGCHVELLFLRYISDWDLDPGRCYRVTWFTSWSPCYDCARHVADFLRGNPNLSLRIFTARLYFCEDRKAEPEGLR
RLHRAGVQIAIMTFKDYFYCWNTFVENHERTFKAWEGLHENSVRLSRQLRRILLPGGSGGSGGSGGSGGSGGSGGIETEQ
EYKEIFITPHQIKHIKDFKDYKYSHRVDKKPNRELINDTLYSTRKDDKGNTLIVNNLNGLYDKDNDKLKKLINKSPEKLLMYHH
DPQTYQKLKLIMEQYGDEKNPLYKYYEETGNYLTKYSKKDNGPVIKKIKYYGNKLNAHLDITDDYPNSRNKVVKLSLKPYRFD
VYLDNGVYKFVTVKNLDVIKKENYYEVNSKCYEEAKKLKKISNQAEFIASFYNNDLIKINGELYRVIGVNNDLLNRIEVNMIDIT
YREYLENMNDKRPPRIIKTIASKTQSIKKYSTDILGNLYEVKSKKHPQIIKKGSGGSTNLSDIIEKETGKQLVIQESILMLPEEVEE
VIGNKPESDILVHTAYDESTDENVMLLTSDAPEYKPWALVIQDSNGENKIKMLSGGSKRPAATKKAGQAKKKKDYKDDDD
KGSGATNFSLLKQAGDVEENPGPMAKPLSQEESTLIERATATINSIPISEDYSVASAALSSDGRIFTGVNVYHFTGGPCAELVV
LGTAAAAAAGNLTCIVAIGNENRGILSPCGRCRQVLLDLHPGIKAIVKDSDGQPTAVGIRELLPSGYVWEG*
