# Supplementary material for: Engineering domain-inlaid SaCas9 adenine base editors with reduced RNA off-targets and increased on-target DNA editing
Source: Nat Commun. 2020 Sep 25;11:4871. doi: 10.1038/s41467-020-18715-y (PMC7519688; doi:10.1038/s41467-020-18715-y)
Supplement: Supplementary file 5 — Description of Additional Supplementary Files [file 41467_2020_18715_MOESM5_ESM.pdf]

File title: Supplementary Data 1
Description: Genomic DNA off-target analysis based on sequence homology of sgRNA targeting ABE1, ABE8, and ABE11. The sgRNA for the top three edited genomic sites were selected and each of the three independent technical replicates were sequenced using parallel mock samples as a negative control (n=3). Data are presented as the sum of reads for each aligned sequence across the three replicates.

File title: Supplementary Data File 2
Description: Amino acid sequence of microABE I744, SaCas9-miniABEmax (V82G), SaCas9-ABEmax, microAIDx I744.
